# Dynamic metabolic interactions and trophic roles of human gut microbes identified using a minimal microbiome exhibiting ecological properties

**DOI:** 10.1038/s41396-022-01255-2

**Published:** 2022-06-18

**Authors:** Sudarshan A. Shetty, Ioannis Kostopoulos, Sharon Y. Geerlings, Hauke Smidt, Willem M. de Vos, Clara Belzer

**Affiliations:** 1 Laboratory of Microbiology, Wageningen University & Research, Wageningen, The Netherlands; 2 Human Microbiome Research Program, Faculty of Medicine, University of Helsinki, Helsinki, Finland; 3 Present Address: University Medical Center Groningen, Groningen, The Netherlands; 4 Present Address: Danone Nutricia Research, Utrecht, The Netherlands

**Keywords:** Microbial ecology, Microbiome

## Abstract

Microbe–microbe interactions in the human gut are influenced by host-derived glycans and diet. The high complexity of the gut microbiome poses a major challenge for unraveling the metabolic interactions and trophic roles of key microbes. Synthetic minimal microbiomes provide a pragmatic approach to investigate their ecology including metabolic interactions. Here, we rationally designed a synthetic microbiome termed **M**ucin and **D**iet **b**ased **M**inimal **M**icrobiome (MDb-MM) by taking into account known physiological features of 16 key bacteria. We combined 16S rRNA gene-based composition analysis, metabolite measurements and metatranscriptomics to investigate community dynamics, stability, inter-species metabolic interactions and their trophic roles. The 16 species co-existed in the in vitro gut ecosystems containing a mixture of complex substrates representing dietary fibers and mucin. The triplicate MDb-MM’s followed the Taylor’s power law and exhibited strikingly similar ecological and metabolic patterns. The MDb-MM exhibited resistance and resilience to temporal perturbations as evidenced by the abundance and metabolic end products. Microbe-specific temporal dynamics in transcriptional niche overlap and trophic interaction network explained the observed co-existence in a competitive minimal microbiome. Overall, the present study provides crucial insights into the co-existence, metabolic niches and trophic roles of key intestinal microbes in a highly dynamic and competitive in vitro ecosystem.

## Introduction

The complexity of interactions within the human gut microbiome contributes to providing health benefits to its host. However, the same complexity presents a major challenge for deciphering metabolic and ecological interactions between the intestinal microbes. Understanding these complex interactions, at both community and individual taxa level, is crucial for the development of effective microbiome modulation strategies [1–3]. The human intestinal tract includes several hundred species mainly belonging to the phyla Actinobacteria, Bacteroidetes, Firmicutes, Verrucomicrobia, Proteobacteria and others [4]. Recently, synthetic microbial communities assembled from host-derived strains have received considerable attention for understanding ecological and metabolic features of the microbiome [5–7]. Synthetic microbial communities of the human intestine can be studied under controlled conditions in vitro [8–13]. In vitro intestinal models allow for stable and controllable conditions as well as frequent sampling of the microbial community that may not be possible with animal models for technical and ethical reasons [14, 15]. Combining in vitro intestinal models with defined microbial communities holds potential for understanding community assembly and structure, compositional and functional dynamics in time and plasticity of microbial interactions.

Studies employing in vitro intestinal models till date have applied either batch, continuous single or semi-continuous or multistage fermentation models [16–20]. An important aspect of the host-associated microbiome is the dietary intake of the host that often follows circadian rhythms and can give rise to stages of excess carbon and energy source and periodic carbon starvation. Both of these aspects may have a profound influence on the compositional and functional dynamics of the microbial community. In fact, previous in vitro studies have revealed that nutrient periodicities can affect microbial community dynamics and physiological functionality [21, 22]. Nutrient periodicity is an important factor that may lead to selection of well adapted taxa, affect microbe–microbe interactions and microbe–environment interactions as well as provide an opportunity for invading species to successfully establish in a community [21–24]. In the human intestinal tract, two major sources of carbon and energy are dietary and host-derived polysaccharides (mainly secreted mucin) that all have a strong deterministic effect on the microbiome [25–27]. The diet can be highly variable on sub-daily time scales posing a major selective pressure on the gut microbiome [28]. Dietary sources, especially complex fiber-derived polysaccharides that reach the colon in a virtually unmodified way, lead to the creation of diverse niches that can support a higher diversity of microbes [29]. In addition, the periodicity and variability in supply of dietary fibers can give rise to dynamic regimes of niche availability consequently affecting interactions between the diet responsive microbes. On the contrary, mucin is a stable source of carbon and energy within a host and is shown to promote stability of the gut microbiome [30]. Therefore, both diet and mucin play a major role in supporting diverse microbial communities and give rise to complex microbe–microbe interactions.

**Fig. 1:**
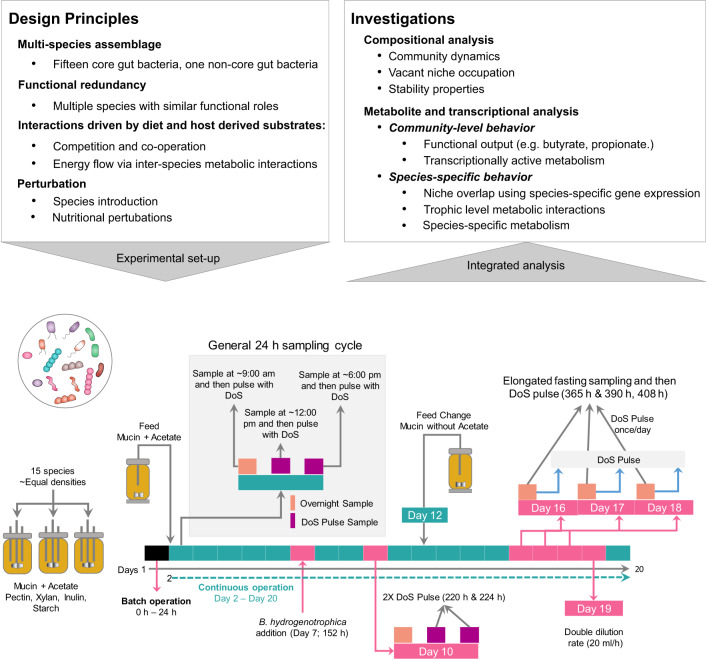
MDb-MM design principles, experiment setup and investigations. Key aspects that were considered when designing the MDb-MM included building a multi-species minimal microbiome with functional redundancy and trophic interactions and potential vacant niches to test niche occupation. The experimental setup included pulse feeding the bioreactors with **D**iet **o**rigin **S**ubstrates (DoS) and introducing perturbations like the addition of new species, increase dietary intake (2X DoS pulse), removal of key metabolite and nutrient starvation. Details about the sampling time points for composition, metabolites and metatranscriptome are depicted in Supplementary Fig. S2.

To understand microbe–microbe interactions within a complex community, it is important to create a community that exhibits ecophysiological properties similar to natural ecosystems [20]. Community-level ecological properties such as resistance and resilience to perturbations, presence of competitors for nutrients as well as mutualists that support metabolic co-operation can be designed in a synthetic minimal microbiome [7]. Here, we sought to investigate microbe–microbe interactions in a synthetic minimal gut microbiome over a period of 20 days under controlled conditions. To explore temporal ecophysiological interactions, the community was assembled in triplicate bioreactors with constant supply of mucin and pulse of the main dietary **D**iet **o**rigin **S**ubstrates (DoS) *viz*. pectin, resistant starch, inulin and xylan. The experiment was designed with various perturbations to test for aspects such as vacant niche occupation by introducing a noncore strain, *Blautia hydrogenotrophica*, increased dietary intake by doubling the concentration of DoS, loss of a key metabolite that is required for growth of specific bacteria by removal of exogenous acetate (coinciding with replenishing of feed medium), diet starvation by subjecting the community to periods of elongated fasting i.e., no addition of DoS for >24 h and increase in substrate feeding rate (Fig. 1). Over a 20-day operation of the artificial gut system, we sampled the three bioreactors at 61 time points each (~3 samples/day) and tested the impact of aforementioned events on the dynamics of MDb-MM composition, structure and function. The integrative analysis of temporal measurements of metabolites, 16S rRNA gene amplicons and metatranscriptomes allowed us to unravel community dynamics and metabolic interactions using a synthetic minimal microbiome.

## Results

### Design of the synthetic Mucin and Diet based Minimal Microbiome (MDb-MM)

We sought to assemble a minimal microbiome that consists of bacterial strains relevant to the human colonic microbiome and mimics key ecological and metabolic properties (Fig. 1). Therefore, the selection of strains was rationally guided by ecophysiological aspects, such as high prevalence (>50%) and minimum abundances threshold of 0.001% in human colonic microbiota, ability to degrade mucin or common multiple dietary polysaccharides that reach the colon in a virtually unmodified form (pectin, xylan, starch and inulin) and their breakdown products. We screened 1155 human gut metagenomes from the curated Metagenomic database and obtained a list of 64 core species (Supplementary Table S1). Majority of these species belonged to Firmicutes (35 species) and Bacteroidetes (25 species). Actinobacteria, Proteobacteria and Verrucomicrobia were represented by five, three and one species respectively. We chose representative strains from Firmicutes, Bacteroidetes, Actinobacteria and Verrucomicrobia. *Bilophila* and *Escherichia* were the two most prevalent genera within Proteobacteria, and we excluded these in this study, because of their low contribution to the overall composition in the human gut metagenomes analysed in this study. Among the core microbiota phyla, Proteobacteria comprised the lowest fraction (1.04%) of the total counts, compared to Firmicutes (46.6%), Bacteroidetes (43.9%), Actinobacteria (6.8%), and Verrucomicrobia (1.4%).

During selection of the candidate strains, we considered competition for growth substrates, known metabolic cross feeding on lactate and 1,2-propanediol (1,2-PD) and the ability to produce lactate or common short chain fatty acids (SCFAs) such as formate, acetate, propionate and butyrate. The details of the representative strains, their known growth substrates and fermentation end products relevant to the current study are given in Table 1.

**Table 1:** General metabolic features of species for which depicted strains were used for MDb-MM.

Species	Strain used/source	Known substrates	Metabolite production^a^	References
*Akkermansia muciniphila*	MucT/ATCC BAA-835	Mucin, N-acetylglucosamine, N- acetylgalactosamine, fucose	A, P, L, 1,2-PD	[12, 31]
*Bacteroides ovatus*	HMP strain 3_8_47FAA	Starch, xylan, inulin	A, P, L, 1,2-PD	[32–35]
*Bacteroides xylanisolvens*	HMP strain 2_1_22	Pectin, starch, xylan	A, P, L	[36]
*Anaerobutyricum soehngenii*	L2-7/DSM 17630	Sugars, DL-lactate, 1,2-PD	B, P, F, CO_2_, H_2_	[37–39]
*Coprococcus catus*	ATCC 27761	Fructose, mannitol, glucose, mannose, lactate	B, P, A, S, H_2_	[40, 41]
*Flavonifractor plautii*	HMP strain 7_1_58FAA	Glucose, maltose, xylose, lysine	L, B, P	[42]
*Eubacterium sireaum*	DSM 15702	Starch, glucose, maltose	A, E, L, B, S	[43, 44]
*Agathobacter rectalis*	DSM 17629	Starch, glucose, lactose, xylose, cellobiose, l-arabinose, trehalose, sorbitol, N-acetylglucosamine	B, A, H_2_, L	[45–47]
*Roseburia intestinalis*	DSM 14610	Starch, glucose, xylose, xylan, arabinose	B, F, L	[48, 49]
*Faecalibacterium prausnitzii*	A2-165	Pectin, inulin, fructose, glucose	B, A, H_2,_ L	[50, 51]
*Subdoligranulum variabile*	DSM 15176	N-acetyl-glucosamine, N-acetyl-mannosamine, cellobiose, dextrin, fructose, fucose, galactose, galacturonic acid, α-glucose, α-lactose, maltose, maltotriose, Mannose, melibiose, rhamnose, salicin, sucrose	B, L, A, S	[52]
*Ruminococcus bromii*	ATCC 27255	Starch, glucose, fructose, galactose	A, F, P, L, E	[53, 54]
*Blautia obeum*	DSM 25238	Arabinose, cellobiose, lactose, mannose, maltose, raffinose, xylose, L-fucose	A, 1,2-PD, P	[55, 56]
*Collinsella aerofaciens*	DSM 3979	Starch, maltose, glucose, sucrose	E, H_2_, A, L; F	[57]
*Bifidoabcterium adolescentis*	L2-32	Inulin, starch, lactose, glucose, xylose, sorbitol, cellobiose, maltose	F, A, L	[46, 58, 59]
*Blautia hydrogenotrophica*	DSM 10507	Cellobiose, lactose, mannose, raffinose, glucose, H_2_/CO_2_, H_2_/formate	A, L	[56, 60]

*A* Acetate, *B* Butyrate, *P* Propionate, *L* Lactate, *F* Formate, *E* Ethanol, *1,2-PD* 1,2-Propanediol, *S* Succinate.

^a^SCFA production varies depending on growth substrates.

### Assembly, co-existence and ecological properties of MDb-MM

**Fig. 2:**
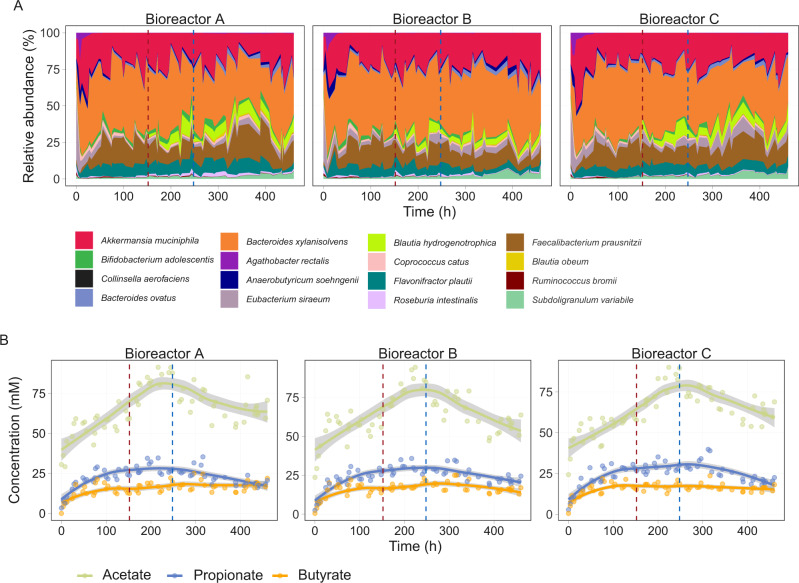
Global response of the MDb-MM. **A** Temporal compositional dynamics of the MDb-MM. **B** Concentration of major SCFAs, acetate, butyrate, propionate produced by MDb-MM in the three bioreactors. The vertical dotted lines, red indicates introduction of *B. hydrogen*o*trophica* (152 h) and blue indicates removal of acetate/feed change (248 h). The curved line represents the locally weighted smoothing (LOESS) for each of the bioreactors and the gray shaded region around these lines shows 95% confidence intervals for the fit. This was calculated and visualized with the default geom_smooth function in ggplot2 R package.

We assembled a MDb-MM consisting of 15 strains representing the core microbiota under batch conditions with both mucin and DoS (Supplementary Table S1 and Supplementary Fig. S2). At 24 h, continuous feed was introduced with mucin and acetate (since some of the strains require it for growth), while DoS were introduced as pulsed feeding thrice daily for the majority of the time points. The species abundance in the MDb-MM was tracked by sequencing of 16S rRNA gene amplicons, and total copies of 16S rRNA genes of the community were quantified using qPCR at 61 time points (Supplementary Fig. S2). The initial 15 species were detected in the three bioreactors for the entire 20-day operation (Fig. 2A). To test vacant niche occupation, the 16^th^ species, *B. hydrogenotrophica* was added at 152 h. At 264 h, the abundance of *B. hydrogenotrophica* was below the amplicon sequencing detection limit. The DoS pulse events resulted in a significant increase in total biomass (optical density; O.D_600_) but this was not captured with total 16S rRNA gene qPCR (Supplementary Fig. S3). No differences in community evenness and number of species contributing to 90% of the total community abundances were detected after DoS pulse events (Supplementary Fig. S4). A steady increase in butyrate, acetate and propionate concentration was observed until the point of removal of acetate from fresh growth media (Fig. 2B). Lactate, succinate, and formate were detected in relatively low concentrations (Supplementary Fig. S5). Formate concentration declined after the removal of exogenous acetate from feed. After 300 h, lactate was not detected in the three bioreactors.

In the first 148 h, before the introduction of disturbances, only propionate was produced in significantly higher concentrations in overnight samples (Wilcoxon test, *p* < 0.001, Supplementary Fig. S6A). The propionate concentration was also significantly higher after addition of *B. hydrogenotrophica* (Wilcoxon test, *p* < 0.001, Supplementary Fig. S6B). However, after the influx of exogenous acetate was stopped, the concentrations of acetate and butyrate were significantly lower in overnight samples (Wilcoxon test, *p* < 0.0001) compared to DoS samples, while propionate production was not significantly affected (Supplementary Fig. S6B). These results demonstrate that the successful assembly of the MDb-MM was achieved in the three bioreactors with presence of the 16 species. The major fermentation end products of MDb-MM were acetate, propionate and butyrate for a period of 460 h. Overall, based on optical densities and metabolite profiles, the MDb-MM was observed to be responsive to DoS pulse feeding as noticed by increase in total biomass (optical density; O.D_600_), and *B. hydrogenotrophica* was able to stably colonize the MDb-MM when introduced into the community after 152 h.

### Temporal dynamics of MDb-MM community

**Fig. 3:**
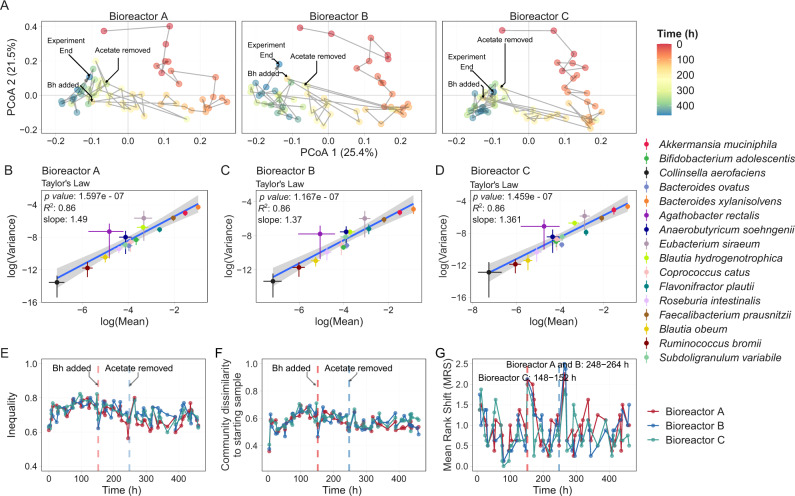
Community-level patterns in MDb-MM. **A** Principal coordinates analysis plot depicting succession of the MDb-MM community over time, community similarity was calculated using Canberra distance. The numbers at each point indicate the time in days of the fermentation experiment. **B**–**D** Power law relationship between variance and mean abundances. The linear regression line is blue and the shaded region represents the confidence interval (geom_smooth function, method = lm). The bars around points represent the lower and upper confidence interval for mean and variance for each of the taxa. **E** Temporal changes in inequality (Gini coefficient) in the community in the three bioreactors. **F** Community divergence based on Canberra distances. **G** Mean rank shift of the MDb-MM in the three bioreactors calculated using the codyn R package.

The MDb-MM showed changes in community structure over time with similar compositions between triplicate bioreactors (Fig. 3A). Recent studies on longitudinal human microbiome data have revealed a linear relationship between log(variance) and log(mean), i.e., species with higher mean abundances tend to also exhibit higher variance in population densities [61–63]. This property is known as the Taylor’s power law [64]. We evaluated whether the MDb-MM assembled in the three bioreactors showed similar time-dependent behavior observed in human gut microbiome [64, 65]. The MDb-MM in the three bioreactors exhibited a linear relationship between log variance and log mean abundance with a slope of 1.45, 1.37, and 1.36 for bioreactor A, B, and C, respectively (Fig. 3B–D). In all three bioreactors, the two most abundant species *Bacteroides xylanisolvens* and *Akkermansia muciniphila* exhibited highest variance while *C. aerofaciens* had lowest variance and was least abundant.

Evenness of species abundances can influence functional stability of microbial communities [66]. We used the Gini coefficient as a measure of evenness, which has values between 0 to 1. Here, 1 indicates a highly uneven community composition [67]. The mean Gini coefficient for the starting MDb-MM at 0 h was 0.62 (±0.01). At the end of the experiment at 460 h, the Gini coefficient for MDb-MM was 0.6, 0.63, 0.62 for bioreactor A, B, and C respectively. The overall mean (± standard deviation) for inequality in MDb-MM was 0.70 ± 0.05, 0.71 ± 0.04, and 0.71 ± 0.05 for bioreactor A, B, and C respectively during the entire experiment.

The long-term divergence of the MDb-MM in all the three bioreactors followed similar trends over time (Fig. 3F). The MDb-MM showed higher deviation from the starting composition during the first phase of the experiment before feed change followed by relatively stable dissimilarities after feed change. Convergence of the three MDb-MM showed similar patterns (Supplementary Fig. S7A). The correlation between community distances and lagged time intervals further supported directional change which was similar in the three bioreactors (Supplementary Fig. S7B). Next, we carried out mean rank shift analysis to identify events when drastic changes occurred in the species ranks (order of relative abundance) within the community. During the initial phase (up to ~100 h) there was a progressive decline in mean rank shift (MRS), but introduction of *B. hydrogenotrophica* caused large fluctuations as did the change of feed with removal of acetate in all three bioreactors (Fig. 3G). The compositional dynamics was highly similar between the three bioreactors (Pearson’s correlation; A and B, *r* = 0.93; A and C, *r* = 0.92; and B and C, *r* = 0.95). These data support highly coherent community-level features of the MDb-MM between the three bioreactors.

### Temporal stability properties of MDb-MM

**Fig. 4:**
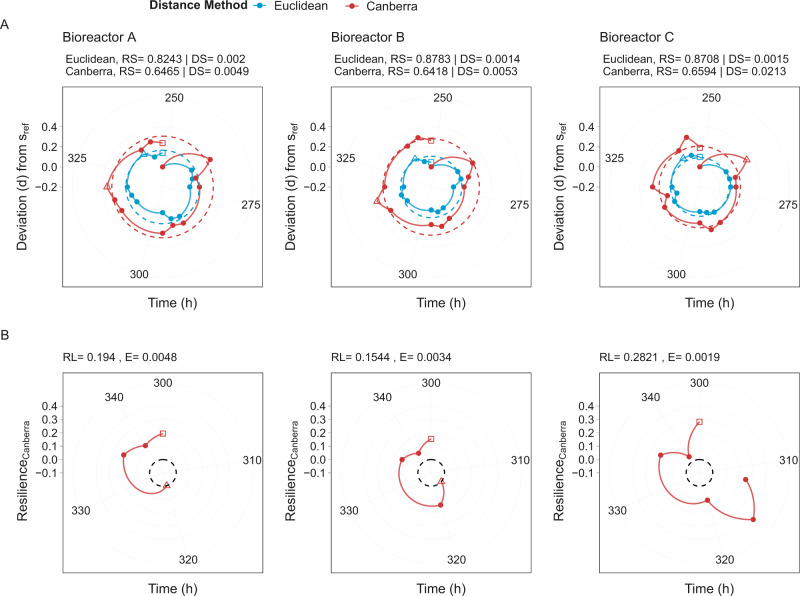
Stability properties of MDb-MM. **A** Community changes from reference phase calculated using Canberra and Euclidean distance. The boundary of reference phase was calculated using the method described by Liu et al. [68]. The shaded region and brown dashed line depict reference phase boundary based on Canberra distance, while the blue dashed line depicts reference boundary based on Euclidean distance. The hollow triangles represent time points when maximal deviation from reference state was observed. **B** Resilience of the MDb-MM after removal of Acetate. The black dashed line depicts reference boundary based on Canberra distance. The stability was calculated with 152 h (introduction of *B. hydrogenotrophica*) as the starting time, removal of acetate/feed change (248 h) as the disturbance event and experiment end point was before elongated fasting was initiated in the three systems (344 h). RS resistance, DS displacement speed, RL resilience.

The observations thus far indicated that the MDb-MM was responsive to the pulse feeding events and perturbation events i.e., addition of *B. hydrogenotrophica* and removal acetate. However, it was unclear if the MDb-MM possesses ecological stability i.e., does the MDb-MM exhibit resistance and resilience to perturbations. To investigate this, we tested the following stability properties of MDb-MM in the three bioreactors [68]: (a) resistance (RS) as the ability of MDb-MM to resist change after perturbations; (b) displacement speed (DS) as the pace at which MDb-MM is displaced upon perturbations; (c) resilience (RL) as the ability of MDb-MM to return to the reference state after a perturbation event, (d) elasticity (E) as the pace at which MDb-MM recovers after displacement due to a perturbation event. The MDb-MM in all three bioreactors exhibited resistance to the change of feed that no longer contained acetate, as for the majority of the time it was observed within the reference state boundary (Fig. 4A, B). In instances where it crossed the reference state boundary, the MDb-MM in all three bioreactors returned to the reference state community (Fig. 4A).

Among the three bioreactors, MDb-MM in C had highest displacement (DS = 0.021) compared to A (DS = 0.004) and B (DS = 0.005), that is deviation from the reference boundary. MDb-MM in bioreactor C also showed highest resilience (RL = 0.282) compared to A (RL = 0.194) and B (RL = 0.154). The larger displacement and resilience values for MDb-MM in bioreactor C suggests the high resilience of MDb-MM and its ability to return to its reference state even after showing the highest deviation in composition [68]. Similar patterns were observed when subsequent perturbation events of elongated fasting and increasing substrate feeding rate from 10 to 20 ml/h were included in the stability analysis (Supplementary Fig. S8A, B). However, the recovery to the reference community state after doubling the substrate feeding rate was on/near the boundary (dashed line, Supplementary Fig. S8A, B) of the reference community state at the end of the experiment.

### Community-level transcriptional activity

**Fig. 5:**
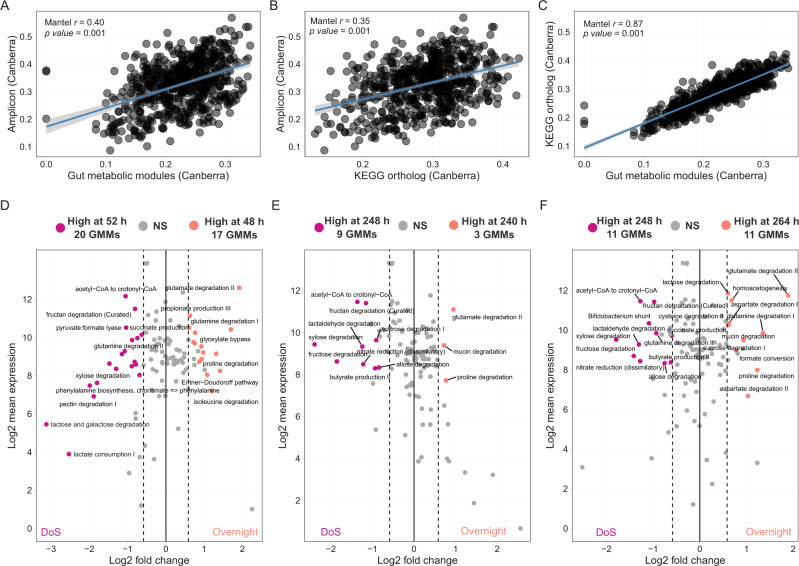
Correlation between compositional and functional succession and transcriptomics response of MDb-MM. Mantel test for correlation between compositional functional community similarity based on Canberra distance. **A** Comparison of community similarity based on 16S rRNA gene relative abundance versus gut metabolic module relative abundances. **B** Comparison of community similarity based on 16S rRNA gene relative abundance versus KEGG ortholog relative abundances. Each circle in these scatter plots represent pairwise Canberra distances between samples. **C** Comparison of gut metabolic module relative abundance versus KEGG ortholog relative abundances. **D**–**F** Differential expression of GMMs in DoS and overnight samples. Before the addition of *B. hydrogenotrophica* with exogenous acetate (48 h vs. 52 h). With *B. hydrogenotrophica* and exogenous acetate (240 h vs. 248 h). With *B. hydrogenotrophica* and without exogenous acetate (248 h vs. 264 h). Modules with adjusted *p* value ≥ 0.01 and with fold change of absolute value ≥1.5 are labeled.

For a subset of the time points, we performed metatranscriptome sequencing. We analysed the transcriptional response at two levels, KEGG orthologs (KOs) as well as gut metabolic modules (GMMs), the latter of which take into account the combination of KOs that are part of specific metabolic modules relevant to the human gut microbiome [69]. The community-level functional divergence using relative abundances of taxa, GMMs and KOs showed similar divergence over time and was linked to changes in the community structure over time (Fig. 5A, Supplementary Fig. S9). Temporal variation in MDb-MM community composition correlated significantly with transcriptional response at both GMM (Mantel_Amplicon vs. GMM_ *r* = 0.40, *p* = 0.001) and KO level (Mantel_Amplicon vs. KEGG_ *r* = 0.35, *p* = 0.001) (Fig. 5A–C). The KEGG and GMM profiles showed good agreement in capturing the temporal variation in MDb-MM gene expression (Mantel_KEGG vs. GMM_ *r* = 0.87, *p* = 0.001). Next, to identify community-level transcriptional response to nutrient periodicity, we compared GMM expression at specific time points (Fig. 5D–F). GMMs linked to carbohydrate degradation were upregulated in the DoS, while mucin and amino-acid degradation were upregulated in overnight samples (Fig. 5D–F). The butyrate production related module “Acetyl-Co-A pathway” was significantly upregulated in the DoS samples (52 and 248 h) in absence of *B. hydrogenotrophica* when exogenous acetate was provided, and after removal of acetate (248 and 264 h) (Fig. 5D–F). In accordance with HPLC data, we observed significantly higher amounts of transcripts encoding enzymes involved in propionate production in overnight samples (48 h) before addition of *B. hydrogenotrophica* (Fig. 5A). After removal of exogenous acetate, there was a significant upregulation of the GMM for formate conversion and homoacetogenesis (264 h), which coincided with an increase in formate concentration observed in metabolite analysis (Fig. 5F and Supplementary Fig. S5).

### Dynamic niche overlap among MDb-MM species

In order to better understand the co-existence of 16 species in the three bioreactors we investigated species-specific metabolic traits. By design, the MDb-MM had multiple species capable of carrying out similar functions—for example, *B. ovatus*, *R. bromii*, *E. siraeum*, and *A. rectalis* can degrade starch (Table 1). Moreover, none of the MDb-MM species were competitively excluded from the system suggesting potential niche partitioning because multiple substrates were available in our system. Therefore, we quantified niche overlap between species in MDb-MM and investigated if there is temporal changes in pairwise species behaviors. We started by calculating the pairwise niche overlap between each of the species at each of the time points for which we had obtained metatranscriptomes. Metabolic module expression was used as quantitative traits for calculating the niche-overlap indices. We used only those GMM traits which are involved in either degradation or consumption of substrates and end-product metabolites (Supplementary Table S2). In this case, a lower niche overlap between species would suggest higher niche segregation and vice versa.

**Fig. 6:**
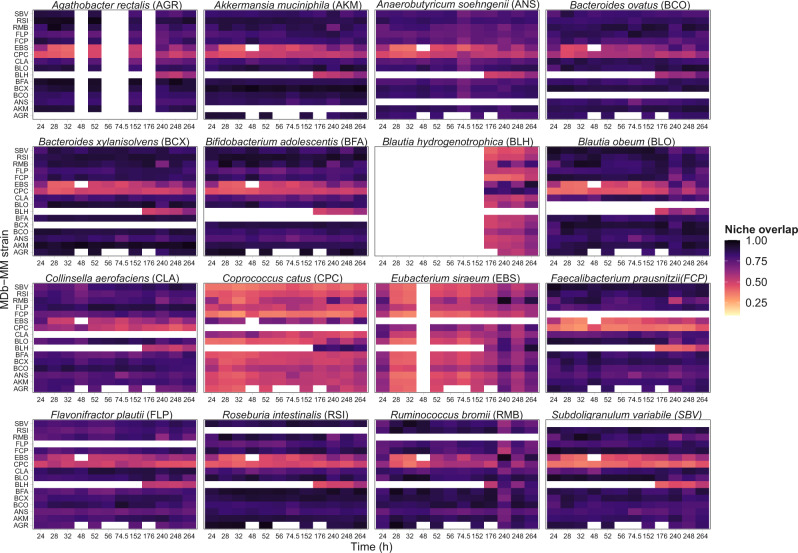
Temporal niche overlap of individual species in MDb-MM. The pairwise niche overlap for each species is plotted as a heatmap with darker color intensity indicate high niche overlap. The abbreviations for species name used on the y-axis are given in brackets of panel headings. The missing values are represented by white color. These are time points when one of the species from the pair had less than 50 counts for GMM traits and hence niche overlap could not be calculated. These are prominent for *A. rectalis and B. hydrogenotrophica. A. rectalis* had one of the lowest 16S rRNA abundances at the initial time points of continuous operation of the bioreactors. *B. hydrogenotrophica* was added at 152 h but RNAseq sample was taken before its addition to the system.

**Fig. 7:**
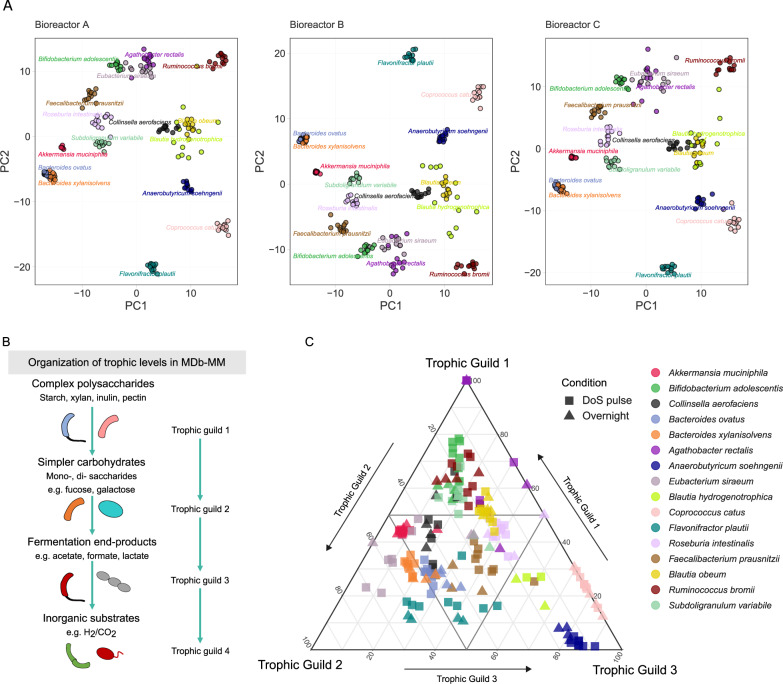
Transcriptional niche segregation and trophic guilds within the MDb-MM. **A** A Principal Component Analysis (PCA) based on GMM trait expression used in trophic guild analysis. The abundances were Hellinger transformed before calculating the Canberra distances. Multiple circles for each species are different time points. The species labels are positioned around the centroids for that particular species. **B** Schematic for organization of metabolic roles into trophic guilds. Trophic guild 1 is for polysaccharide and mucin degradation, trophic guild 2 consists of mono-di-saccharides trophic guild 3 consists of consumption of fermentation ends/by-products and trophic guild 4 consists of those consuming inorganic substrates for growth. **C** Ternary plot indicates the trophic status of the minimal microbiome strains at different time points. For every strain at a given time point, we summed its expression and calculated the relative expression for each trophic guild. The proximity of the symbols to the apex of the triangle is proportional to the averaged potential contribution of each strain to trophic guilds. The trophic guild 4 is not shown in this figure. The ranking of species within each trophic guild is provided in Supplementary Fig. S11.

All species demonstrated temporal variation in niche overlap with other species in MDb-MM, highlighting the dynamic nature of inter-species interactions in the MDb-MM (Fig. 6). Comparison of pairwise distributions of niche-overlap values revealed that the complex substrate degraders, *B. xylanisolvens*, *A. muciniphila*, *A. rectalis*, *B. adolescentis*, *S. variabile*, *F. prausnitzii*, and *R. bromii* showed comparatively higher niche overlap (>0.75) with each other (Supplementary Fig. S10). *C. catus*, *A. soehngenii* and *E. siraeum* often had the lowest niche overlap with the other strains in the community. For some of the time points, *A. rectalis* had low number of transcripts for several of the GMM traits and we were unable to measure pairwise niche overlaps. We then compared the overall expression of GMM traits for all species at different time points and observed niche segregation based on transcriptional responses of metabolic pathways consistently in the three bioreactors (Fig. 7A). The two *Bacteroides* species exhibited low niche segregation and *C. aerofaciens* and the two *Blautia* species were closely located on the two-dimensional ordination plot. *C. catus*, *A. soehngenii* and *F. plautii* had distinct transcriptional patterns. These data suggest that the observed co-existence likely resulted from each species occupying a specific metabolic niche and that inter-species cross-feeding supported non-complex substrate degraders forming a trophic interaction network.

### Trophic guilds and niches of MDb-MM species

The metabolic flow and biomass distribution within the gut is largely driven by bacteria with specialized molecular machineries capable of degrading complex carbon sources [70] The action of polysaccharide degraders (primary consumers) results in niche construction that may be dependent on the source substrate as well as their metabolic pathways. Consequently, this leads to formation of a hierarchal organization within the community into trophic levels [70]. Here, based on metatranscriptomic species-level assignment of transcriptional expression of GMMs, we broadly classified them into four trophic guilds similar to those reported previously from computational simulations [70] (see Fig. 7B and methods). Transcriptional contribution of species to each of the trophic guilds revealed the inter-species connectedness of resource utilization.

Ranking of MDb-MM strains based on the relative proportions of their GMM expression within each trophic guild revealed temporally changing trophic roles (Supplementary Fig. 11). This suggested that trophic roles are dynamic in MDb-MM. In addition, these observations also suggested that transcriptional expression of individual species for each of the trophic level can be variable. Furthermore, to investigate whether the trophic role is associated with abundance of species in the community we compared the relative abundance of species and its ranking within a trophic guild. We observed that bacteria that are dominant in trophic guild 1 had higher abundances while those dominating trophic guilds 3 or 4 had lower relative abundances in the MDb-MM (Supplementary Fig. 12). This suggests that the species dominating trophic guild 1 are usually present in higher abundances in microbiomes.

The two most abundant species in MDb-MM (Figs. 1D, 5A), *A. muciniphila* and *B. xylanisolvens*, contributed to two trophic guilds: degradation of complex substrates i.e., trophic guild 1 and degradation of simpler carbohydrates i.e., trophic guild 2 (Fig. 7). Known starch degraders, *R. bromii*, *B. ovatus*, *C. aerofaciens*, *E. siraeum*, and *A. rectalis* showed transcriptional segregation across the trophic guild 1 and 2 axis. *S. variabile*, *B. adolescentis* and *R. bromii* dominated trophic guild 1 and showed metabolic activity for arabinoxylan, fructan and starch degradation, respectively (Supplementary Fig. S13).

The action of species occupying trophic guild 1 can give rise to extracellular mono- and di-saccharides that can be utilized by species that lack specialized molecular machineries for polysaccharide degradation. In our system, breakdown of mucin, pectin, inulin, starch and xylan could result in simple mono- and di-saccharides such as fucose, galactose, galacturonate, fructose, maltose or xylose as major simple carbohydrates. Within trophic guild 2, fucose transport and degradation genes were identified to be transcribed in *A. muciniphila* and *B. obeum* (Supplementary Fig. S14). In addition, transcription of galactose metabolism genes was predominantly detected in *A. muciniphila*, *B. ovatus* and *B. xylanisolvens*. Galacturonate is the main component in pectin, and *F. prausnitzii* and to some extent *B. ovatus* and *B. xylanisolvens* were found to express genes involved in its degradation (Supplementary Fig. S14).

We classified consumption of fermentation end products such as acetate, lactate, 1,2-PD and formate as trophic guild 3. These are mostly major end products of carbohydrate fermentation, while utilization of H_2_ and CO_2_, inorganic by-products of acidogenesis, are classified here as trophic guild 4. Specialist trophic guilds could be assigned to *A. soehngenii*, *B. hydrogenotrophica*, and *C. catus* as their transcriptional activity was largely contributing to trophic guild 3 (Fig. 7 and Supplementary Fig. S15). *F. plautii* showed variation across trophic guild 2 and 3. In our experimental setup, acetate was exogenously supplied until 248 h to the MDb-MM and then removed from the feed. Expression of modules for acetate to acetyl Co-A via I and II (acetate kinase pTKA) was observed in *A. soehngenii*, *F. prausnitzii*, *B. obeum*, *B. hydrogenotrophica*, and *F. plautii* (Supplementary Fig. S15). *A. soehngenii* and *F. prausnitzii* are known to have improved growth in the presence of acetate, which would explain the activity for consuming acetate [50, 71]. Cross-feeding of lactate resulting from the metabolism of polysaccharide degraders such as *Bifidobacterium* and *Lactobacillus* by butyrate producers in the human gut is well known [71, 72]. Here, we detected very low amounts of lactate in the metabolite analysis which resembles the situation in fecal samples where lactate is hardly detected [71]. This can be explained by the significant transcriptional activity for lactate consumption primarily *via* the *lctABCDE* pathway (Supplementary Fig. S14). *A. soehngenii* showed high transcriptional activity for utilization of lactate plus acetate, which further confirms our previous observation of this being a specialized niche for this organism [20, 73]. *C. catus* demonstrated activity for lactate consumption but is known only to consume the L-form of lactate, while *A. soehngenii* can use both the D- and L-forms of lactate [74]. Fucose fermentation results in production of 1,2-PD, which is another well-known cross-feeding metabolite [37, 72]. While we did not detect any 1,2-PD, there was higher transcriptional activity for utilization of 1,2-PD in *A. soehngenii* compared to *B. obeum*, which also produces propionate (Supplementary Fig. S10) [16, 41]. Transcriptional activity for autotrophic growth on H_2_ and CO_2_ using formate dehydrogenase and formate-tetrahydrofolate ligase was observed in *B. hydrogenotrophica*. Other than CO_2_ and H_2_, we observed active processes for dissimilatory nitrate and sulphate metabolism within guild 4. Among the two *Bacteroides* species, *B. xylanisolvens* was the dominant species in the MDb-MM and had higher contribution to trophic guild 4, which was observed to be linked to higher expression of the nitrate reduction module. Dissimilatory nitrate reduction to ammonium may be an advantageous strategy for higher growth rate in competitive ecosystems. In summary, the 16 species in the MDb-MM co-existed by occupying and interacting at different trophic levels to form a complex web of inter-species interactions.

## Discussion

Due to technological and practical limitations, deciphering the community dynamics and microbe–microbe interactions is challenging using fecal or other intestinal samples derived from human. Here, we investigated microbe–microbe and microbe–environment interactions at species and community level within a highly controlled setting, using a defined microbiome that we subjected to detailed compositional, transcriptional and metabolic analysis. The three most important aspects of this study are (i) assembly of a human minimal microbiome that exhibits ecologically relevant interactions, (ii) the experimental setup which included nutrient periodicity and (iii) a set of specific biotic and abiotic perturbations that allowed to address the resilience of the system. All of these aspects are crucial for better understanding the interactions dynamics within human intestinal microbial communities [5, 7]. Our rational selection was largely driven by understanding of the anaerobic physiology of key human gut microbes. Knowledge of microbial physiology was complemented by considering ecological aspects at the community-level such as assembly, co-existence, competition for resources and cross-feeding. This enabled us to first demonstrate the applicability of ecological concepts, e.g., Taylor’s law, community turnover, divergence, resistance and resilience, and then to investigate the species-level metabolic interactions using metatranscriptomics [2, 9, 75–79]. The MDb-MM exhibited significant correlation with respect to dynamics of composition, metabolic output and transcriptional response in replicate bioreactors. This supported previous observations in synthetic microbiomes that a common pool of species shows similar/reproducible assembly and community-level dynamics under similar growth condition and exposure to similar perturbation events [80–82]. This is equivalent to the classical enrichment experiments where the emergent community assembly can be driven by selecting for specific bacteria or consortia with specific substrates and/or environmental factors such as high salt, pH or temperature [83]. Future research is warranted to test whether a different combination of species than the one used here, would result in similar community-level behaviors under identical perturbations [24, 81]. Additionally, modeling of synthetic microbiota based on complementary wet lab experiments can further increase our understanding of interactions and dependencies in the intestine [80, 84, 85]. Nonetheless, we demonstrated how ecophysiology guided design of synthetic minimal microbiomes combined with metatranscriptomics is a promising avenue to investigate core concepts in ecology and unravel potential metabolic interactions.

At individual taxa level, we observed highly variable compositional and functional responses. This could be attributed to potential technical variation in measurements and/or deterministic chaos [86, 87]. At community-level the behavior can be rather deterministic as observed with similar divergence, mean rank shift and inequality in triplicate MDb-MMs when subjected to similar external perturbations [81, 88, 89]. It is, however, important to note that our system was highly controlled with only one event of immigration (addition of *B. hydrogenotrophica*) and stochastic processes such as dispersal limitation not being enforced in our experimental setup [90]. Nevertheless, our observation of deterministic assembly of MDb-MM has some implications for designing microbiome modulation strategies, where achieving community-level stability in both composition and function may be crucial. Examples are resistance to invasion or enhanced butyrate production, which can be achieved by targeting ecosystem level properties using appropriate prebiotics [1, 91, 92]. These prebiotics may not necessarily target a specific species but a group of species whose fundamental niche allows for “insurance” to absorb the impact of daily stochastic and destabilizing forces [79, 93].

The investigation of species-specific transcriptional responses revealed that the core gut microbes used in this study have highly evolved metabolic strategies which could explain their co-existence with other seemingly competitive core species. The co-existence is likely due to the ability of these core gut microbes to dynamically regulate the transcriptional response for utilizing specific carbon and energy sources that are vacant [29, 78]. This allows individual species to occupy the niches that become available over time either, due to external (inflow of diet) or changing metabolic behavior of competitor species. For instance, we observed, at transcriptional level, changing patterns of polysaccharide utilization among the species that are part of the first trophic guild where no single species dominated transcriptional contributions for the entire duration of the experiment. These observations provide support for the role of “functional insurance” as result of the presence of competitive species in maintaining community composition, structure and functional stability.

Another aspect of host-associated microbial communities is the immigration of new species which can have an impact on the overall community [94, 95]. By introducing *B. hydrogenotrophica* in the established minimal microbiome, we demonstrated a widely appreciated role of vacant niches in supporting survival of immigrating species [29, 96]. Despite its fundamental niche being diverse including the ability to utilize several simple carbohydrates that were available, *B. hydrogenotrophica* likely utilized H_2_/CO_2_ and/or formate as observed with active expression of the formate conversion module [56]. When we removed exogenous acetate, butyrate production declined, and this can be attributed to the fact that acetate is one of the key metabolites for its production. Importantly, after removal of exogenous acetate, *B. hydrogenotrophica* showed high expression of modules linked to homoacetogenesis thus highlighting its contribution to acetate production. This could have aided in stabilizing the community because butyrogenic species such as *A. soehngenii*, *F. prausnitzii* and *R. intestinalis* require acetate for improved growth. This highlights the potential for cyclic interactions where end products of lower trophic guilds can help species occupying higher trophic guilds. Overall, these data provide support for a specialized niche of *B. hydrogenotrophica* that includes inorganic substrates and/or formate [8, 11, 78]. *B. hydrogenotrophica* can be considered a key species, which can potentially support production of butyrate. For instance, enhancing butyrate production *via* prebiotics can lead to significant amounts of gases and therefore recycling these into acetate by autotrophic acetogens such as *B. hydrogenotrophica* can further support butyrate production in a trophic network with butyrate producers [71].

The flow of energy in biological ecosystems is widely described via trophic structures where energy flows from one level to another [70, 97]. The so-called keystone species are usually defined for taxa at higher trophic levels [54, 98]. Our analysis highlights the difficulties in assigning strict hierarchy based on single and specific trophic roles for individual taxa, especially because the breakdown of complex substrates results in simpler substrates, which the primary degrader can also utilize. Furthermore, the temporal differences we observed in dominance of each bacterium within the trophic guilds indicates that functional roles of bacteria can vary over time within a community. We observed certain taxa with a prominent role within specific trophic guilds. For instance, *A. soehngenii* and *C. catus* were predominantly part of the trophic guild level 3 which involves consuming fermentation end products, lactate and 1,2-PD. This observation further supports our previous findings that *A. soehngenii* occupies an energetically challenging niche, i.e., the consumption of lactate and acetate [20]. In contrast, *B. hydrogenotrophica* occupied the lowest trophic guild consuming inorganic substrates. Thus, MDb-MM allowed us to unravel functional roles of each of the key gut species in presence of other core microbiota. In addition, we were able to identify potential metabolic interactions and cross-feeding occurring within the MDb-MM by investigating trophic guilds associations based on species-specific transcriptional profiles for GMMs related to degradation of complex substrates, production and consumption of fermentation products like formate and lactate.

Our experimental system did not take the host-aspect into account, which will influence the community composition and dynamics [99]. Hence, improvements can be envisaged by incorporating the MDb-MM in an in vitro model such as HUMix and organoid cell cultures [15, 100, 101], that comprise host features such as aspects of the immune system. The ability to track abundances of closely related species across time points in synthetic communities is crucial. Here, we used short amplicons of the V5-V6 (~280 bp) region of the 16S rRNA gene and noticed non-specific amplification of *B.hydrogenotrophica* at few time points prior to its addition. In such scenarios, using whole shotgun metagenomics might provide better resolution. One of the major challenges we faced during this study was the difficulty in predicting the metabolic functions based simply on automated annotation and analysis. For instance, the identification of an amylase gene (K01176, alpha-amylase [EC:3.2.1.1]) with high expression in *A. muciniphila* suggested its contribution to starch degradation. This gene is likely coding for a glycoside hydrolase involved in breaking glycosidic linkages present in mucin and is not involved in starch degradation. These observations highlight the need for careful curation and interpretation of -omics based functional analysis of fecal samples where the majority of the species remain uncharacterized. With some manual curation of the published GMMs, we were able to capture >87% of the variation between samples that were identified at KO level annotation. This suggests that it is also valuable to investigate other key functions such as those involved in signaling and processing, virulence, vitamin and co-factor biosynthesis and their role in the species dynamics we observed in this study. We did not include bile salts in our media, and several key vitamins and co-factors such as vitamin B_12_ were provided exogenously. Therefore, impact of these key compounds on the community remains unknown. In addition, a bioreactor with similar setup but with constant supply of DoS could help in identifying if the pulse feeding played a role in co-existence of all species till the end of the experiment.

In this study, we created a minimal microbiome that exhibits ecological stability properties and intricate metabolic interactions that are observed in more diverse and complex natural ecosystems. We provide experimental evidence for temporally variable niche occupation as one of the important mechanisms by which species competing for similar resources can co-exist in a dynamic ecosystem. In addition, we demonstrate how metatranscriptomics can be used to assign quantitative traits for identifying niche overlap at transcriptional level. We foresee the use of data generated in this study to serve as a useful resource for ecologists, systems biologists and microbiome experts for developing predictive ecological and metabolic models and improving our understanding of the human gut microbiome.

## Materials and methods

### Species selection for the composition of the synthetic MDb-MM

Taxonomic composition data from metagenomic studies was obtained from the curatedMetagenomicData data package (v1.18.2) [102]. To identify the taxa that are part of the core microbiota we analysed species-level data from 1155 “Western healthy” human gut metagenomes covering general populations from North America and Europe. A total of 64 metagenomic species, which were present in at least 50% of all samples were analysed with a minimum relative abundance of 0.00001 [103].

### Bacterial strains used in this study

The following strains were obtained from the German Collection of Microorganisms and Cell Cultures (DSMZ, Braunschweig, Germany) or the American Type Culture Collection (ATCC, Manassas, USA): *Agathobacter rectalis* (DSM 17629), *Eubacterium siraeum* (DSM 15702), *Roseburia intestinalis* (DSM 14610), *Subdoligranulum variabile* (DSM 15176), *Blautia obeum* (DSM 25238), *Blautia hydrogenotrophica* (DSM 10507), *Coprococcus catus* (ATCC 27761), *Ruminococcus bromii* (ATCC 27255), and *Collinsella aerofaciens* (DSM 3979/ATCC 25986). *Anaerobutyricum soehngenii* (DSM 17630, L2-7) was kindly provided by Prof. Harry J. Flint’s group (University of Aberdeen, UK). The strains from the human microbiome project (HMP) catalog were *Bacteroides* sp. 3_8_47FAA (*Bacteroides ovatus*), *Bacteroides* sp. 2_1_22 (*Bacteroides xylanisolvens*) and *Flavonifractor plautii* 7_1_58FAA. Furthermore, *Akkermansia muciniphila* (ATCC BAA-835), *Bifidobacterium adolescentis* (L2-32), and *Faecalibacterium prausnitzii* (A2-165) were taken from the culture collection of the Laboratory of Microbiology, Wageningen University & Research, The Netherlands.

### Medium composition for MDb-MM strains

All strains were grown in a medium with the following composition: KH_2_PO_4_ (0.408 g/L), Na_2_HPO_4_.2H_2_O (0.534 g\L), NH_4_Cl (0.3 g/L), NaCl (0.3 g/L), MgCl_2_*6H_2_O (0.1 g/L), NaHCO_3_ (4 g/L), yeast extract (2 g/L), beef extract (2 g/L), CH_3_COONa (2.46 g/L), casitone (2 g/L), peptone (2 g/L), cysteine-HCl (0.5 g/L), carbohydrates (1.1 g/L), resazurin (0.5 mg/L), 1 mL trace elements in acid (50 mM HCl, 1 mM H_3_BO_3_, 0.5 mM MnCl_2_*4H_2_O, 7.5 mM FeCl_2_*4H_2_O, 0.5 mM CoCl_2_, 0.1 mM NiCl_2_ and 0.5 mM ZnCl_2_, 0.1 mM CuCl_2_*2H_2_O), 1 mL trace elements in alkaline (10 mM NaOH, 0.1 mM Na_2_SeO_3_, 0.1 mM Na_2_WO_4_ and 0.1 mM Na_2_MoO_4_), 1 mL hemin solution (50 mg hemin, 1 mL 1 N NaOH, 99 mL dH_2_O), 0.2 mL vitamin K1 solution (0.1 mL vitamin K1, 20 mL 95% EtOH). After autoclaving and before inoculation, 1% of vitamin solution was added (11 g/L CaCl_2_, 20 mg biotin, 200 mg nicotinamide, 100 mg p-aminobenzoic acid, 200 mg thiamin (vitamin B_1_), 100 mg panthothenic acid, 500 mg pyridoxamine, 100 mg cyanocobalamin (vitamin B_12_), and 100 mg riboflavin). This basal medium composition was used for both pre-cultures and the bioreactors and the feed with differences in carbon source supplementation.

For pre-cultures, the bacteria were grown in serum bottles in anoxic conditions with 80/20 CO_2_/N_2_ as mixed gas using different combinations of carbon sources (Supplementary Table S3). The pre-cultures were incubated non-shaking at 37 °C for 24 h.

### Anaerobic bioreactor operation

Fermentations were conducted in three parallel bioreactors (DasGip, Eppendorf, Germany) filled with 300 ml of the abovementioned growth medium at 37 °C, at a stirring rate of 100 rpm. For the first 24 h, the bioreactors were operated in batch mode where the 300 mL growth medium was supplemented with 5 g/L mucin from porcine stomach type III (Sigma-Aldrich) as well as Diet origin Substrates (DoS) which comprised of 1.11 g/L of each of xylan (beechwood, Apollo scientific, U.K.), soluble starch (from potato) (Sigma-Aldrich, USA), inulin (from chicory) (Sigma-Aldrich, USA) and pectin (from apple) (Sigma-Aldrich, USA) at the beginning of the fermentation. The carbon sources, except for mucin, were prepared as 60 g/L stock solutions. These stock solutions were prepared anoxically in serum bottles and autoclaved prior to adding the carbon sources to the bioreactors. The pH was controlled at 6.8.

The bioreactors were inoculated with a normalized O.D. of 1.0 of each one of the abovementioned species in order to have the same cells abundance at the beginning of the fermentation. A single inoculum mix was prepared from the same pre-cultures. The three bioreactors thus represent technical replicates for a single experiment. This was done to avoid potential technical errors in preparation of starting inoculum which may influence the behavior of species within the community resulting in inter-bioreactor differences. After allowing the species to grow for 24 h, continuous operation of the bioreactors was initiated. The flow rate of the feed was set to 10 mL/h with a medium retention time of 30 h. In our experiment we used a retention time of 30 h, which is within the range of gut transit times [104–106].

In the first phase of continuous feed supply i.e., from 24 h up to 248 h, basal medium in the feed consisted of 5 g/L mucin and 30 mM of sodium acetate. During the continuous operation, the bioreactors were spiked three times a day with a 4 h gap with DoS (xylan, soluble starch, inulin and pectin) with a final concentration of 1 g/L in each bioreactor. After the first 24 h, we initiated pulsed feeding of DoS and sampled for metabolite and 16S rRNA gene analysis as follows: The 24 h sample taken at ~9:00 h represented overnight sample and after sampling the bioreactors were pulsed with DoS and the community allowed to grow undisturbed until ~13:00 h. At this time, we collected samples for analysis and immediately following this a second DoS pulse was introduced. We then allowed the MDb-MM to grow until ~17:00 h at which point we sampled again. This represented the second DoS pulse sample of the day. This was followed by a third DoS pulse, the MDb-MM grew overnight, and the next day at 9:00 h we sampled to repeat the cycle of sample-pulse-grow-sample-pulse. At 248 h of bioreactor operation, we replenished the feed with freshly prepared anoxic growth medium but this time we removed sodium acetate and only 0.5% mucin was added.

During the fermentation period (2 weeks) different perturbations were introduced in the system. These disturbances included the addition of *Blautia hydrogenotrophica*, the increase of the concentration of carbohydrates addition to 2.22 g/L, elongation of the fasting period from 16 to 21 h, increase of the substrate feeding rate to 20 ml/h. These events are depicted in Supplementary Fig. 2. Samples were taken during both the fasting and feeding period and at every perturbation point (Schematic overview Fig. 1 and Supplementary Fig. 2). Samples for DNA and HPLC were stored at −20 °C. Samples for RNA were centrifuged at 4816 × *g* for 30 min at 4 °C. Then, 1 mL of RNAlater was added to the pellet, the pellets were snap-frozen in liquid nitrogen and stored at −80 °C.

### High performance liquid chromatography (HPLC)

For fermentation product analysis, samples were obtained at different time points of the incubation period. Crotonate was used as the internal standard, and the external standards were lactate, formate, acetate, propionate, butyrate, isobutyrate, 1,2-PD, sialic acid and glucose. Standards were prepared in the following concentrations: 2.5, 5, 10, and 20 mM. Substrate conversion and product formation were measured with Shimadzu LC_2030C equipped with a refractive index detector and a Shodex SH1011 column. The oven temperature was set at 45 °C with a pump flow of 1.00 mL/min using 0.01N H_2_SO_4_ as eluent. All samples and standards (10 µl injection volume) ran for 20 min.

### DNA isolation and library preparation

Genomic DNA was extracted using the FAST DNA Spin kit (MP Biomedicals, Fisher Scientific, The Netherlands) following the manufacturer’s instructions. We included positive controls, a mock community DNA with known composition [107] and reagent controls for DNA extraction and PCR. The concentration of genomic DNA was measured fluorometrically using Qubit dsDNA BR assay (Invitrogen). The hypervariable region V5-V6 (~280 bp) of the 16S rRNA gene was amplified with Phusion Hot Start II DNA polymerase (2 U/μL) for 25 cycles using 0.05 μM of each primer (784F–1064R) that both contained sample-specific barcodes at their 5′-end. The amplification program for PCR included an initial step of 98 °C for 30 s, then 25 cycles of at 98 °C for 10 s, followed by an annealing step at 42 °C for 10 s and elongation step at 72 °C for 10 s and a final extension at 72 °C for 7 min. PCR products were purified using MagBio beads according to the manufacturer’s protocol. Purified products were quantified using Qubit dsDNA BR assay kit (Life Technologies, USA) and were pooled in equimolar amounts into one single library. After pooling, the mixed libraries were concentrated using MagBio beads to a concentration needed by the sequencing company. The samples were sequenced on a NovaSeq platform (Illumina) in 2 × 150 base paired-end mode at Novogene (U.K).

### qPCR

The total abundance of all species in the synthetic community was determined by qPCR. The DNA concentrations were measured fluorometrically (Qubit dsDNA BR assay, Invitrogen) and adjusted to 1 ng/μL by diluting them in DNase/RNase-free water and prior to use as the template in qPCR. Universal primers targeting the 16 S rRNA gene of all the species (1369F 5′-CGG TGA ATA CGT TCY CGG-3′ and 1492R 5′-GGWTACCTTGTTACGACTT-3′; 123 bp) were used for quantification. A standard curve targeting the 16 S rRNA gene of *B. thetaiotaomicron* was prepared with nine standard concentrations from 10^0^ to 10^8^ gene copies/μL. The qPCR was performed in triplicate with iQ SYBR green supermix (Bio-Rad, USA) in a total volume of 13 μL prepared with primers at 500 nM in 384-wells plates with the wells sealed with optical sealing tape. Amplification was performed with an iCycler (Bio-Rad): one cycle of 95 °C for 5 min; 40 cycles of 95 °C for 15 s, 60 °C for 20 s and 72 °C for 30 s each; one cycle of 95 ^o^C for 1 min; and a stepwise increase of temperature from 60 to 95 °C (at 0.5 °C per 5 s) to obtain melt curve data. Data were analysed using CFX Manager 3.0 (Bio-Rad).

### RNA isolation

The cells (10 mL) were centrifuged at 4816 × *g* for 15 min at 4 °C and the supernatant was discarded. Total RNA was isolated by combining enzymatic lysis, the Trizol reagent and the RNeasy mini kit (QIAGEN, Germany). A mixture of lysozyme (15 mg/mL), mutanolysin (10 U/mL) and Proteinase K (100 µg/mL) in 1X TE buffer was added to the pellet normalizing to an OD600 of 2.0 per 100 µL of this mixture. The samples were mixed by vortexing and incubated at room temperature for 10 min. After 5 min of incubation, the samples were vortexed again. Four microliters of p-mercaptoethanol mixed with 400 µL RLT buffer was added to the sample. Subsequently 1 mL of Trizol reagent was added to 100 µl of the sample. This mixture was transferred to a sterile tube containing 0.8 g of glass beads (diameter of 0.1 mm). The tubes were homogenized by bead beating three times for 1 min at 5.5 m/s, while cooling the samples on ice in between steps (bead beater, Brand). Then, 200 µL of ice-cold chloroform was added. The tubes were mixed gently and centrifuged at 12,000 × *g* for 15 min at 4 °C. The RNA isolation was continued following the manufacturer’s instructions of the RNeasy mini kit, including an on-column DNase step using DNase I recombinant, RNase-free, (Roche Diagnostics, Germany) incubating at 37 °C for 30 min. RNA concentration was measured using Qubit and the quality was determined by the Qsep100 bioanalyzer (BiOptic inc, Taiwan). The RNA samples were stored at −80 °C until further processing. Further processing such as removal of rRNA, library preparation and sequencing was performed by Novogene using platform NovaSeq PE150 (Illumina).

### Bioinformatics

#### Amplicon data analysis

The 16S rRNA gene amplicon sequencing data was analysed using the DADA2 R package [108]. Raw data (total 4,27,03,796 reads) was filtered to remove low quality reads and reads with more than 2 errors and those matching the PhiX (filterAndTrim function) resulting a total of 4,18,65,602 reads which were then subjected to removal of chimeric sequences (removeBimeraDenovo, consensus method), an average of 225083 ± 102107 reads per samples were obtained (Supplementary Table S4). We used a custom database consisting of 16S rRNA gene sequences fetched from the genomes of the 16 bacterial strains used in this study using barnap (available at https://github.com/mibwurrepo/Shetty_et_al_MDbMM16) [109]. On average 97 ± 1.9% of the reads were assigned to the MDb-MM strains (Supplementary Table S4). Taxonomic assignment was done using the RDP classifier [110]. The unique amplicon sequence variants (ASVs) were merged at species-level using the *tax_glom()* function in phyloseq (v1.32) [111]. The species counts were normalized for the differences in 16S rRNA gene copy number (Supplementary Table S3) and absolute counts were calculated as described previously [112]. Further analysis of the community composition and structure was done using the microbiome R package (v.1.10.0) [113]. Data visualization packages, ggplot2 and ggpubr R packages were used for plotting figures [114, 115].

#### Metatranscriptomics analysis

A total of 816,752,875 raw paired-end reads totaling to 244.9 giga base pairs were obtained from thirty-six samples (Supplementary Table S5). We followed the approach described in the SAMSA2 pipeline [116]. The forward and reverse adaptors were filtered using Trimmomatic (v0.36) (settings: PE -phred33, SLIDINGWINDOW:4:15, MINLEN:70) and then merged using pear (v0.9.10) [117, 118]. Merged reads matching the ribosomal rRNA were removed with SortMeRNA (v2.1) [119]. A custom database was created from genome sequences of all the bacterial strains used in this study. All the genome sequences in FASTA format were downloaded from the NCBI genome database. For consistency all the genomes were re-annotated using Prokka (v1.12) and the 16S rRNA gene copy numbers for individual strains were identified using the barrnap (v0.9) tool [109, 120]. The amino-acid sequences from each strain were then combined to create a database compatible with DIAMOND (v 0.9.22.123) using the *makedb* function [121]. The ribosomal sequences depleted reads were annotated with DIAMOND using blastx. The DIAMOND output files were further analysed in R. The corresponding codes are available at (https://github.com/mibwurrepo/Shetty_et_al_MDbMM16). The amino-acid sequences obtained from genomes were also annotated using the KEGG databases using the GhostKola tool for KEGG ortholog (KO) annotations [122].

#### Gut metabolic modules (GMMs)

We did additional curation for the metabolic modules from our previous study to incorporate further refinements for the strains used in this study [20]. The curated GMMs are available at the GitHub repository of this study (https://github.com/mibwurrepo/Shetty_et_al_MDbMM16). We used counts per million normalized KO abundances (*cpm* function in edgeR R package v3.24.3) for profiling the metabolic modules using the omixer-rpmR R package (v0.3.1) [123, 124]. The parameters for the *rpm* function in omixer-rpmR, were as follows, score.estimator = “median”, contribute = 0.5, KO = 2, distribute = NULL.

#### Niche overlap and trophic organization

A lower niche overlap (NO) would suggest higher transcriptional niche segregation and vice-versa between species. We used the NO index using the kernel density estimates approached described by Mouillot et al. [125]. The function to calculate niche overlap was adapted from here https://github.com/umr-marbec/nicheoverlap/blob/master/nicheoverlap.R This niche-overlap index is non-parametric and assumes no normality in trait values. We used the GMM framework in which we used metabolic module expression as quantitative traits for calculation of niche-overlap index. A schematic figure depicting the calculation approach is shown in Supplementary Fig. S16. We calculated pairwise niche overlap using the species GMM trait abundances for each of the time points separately as the area of overlap between the density distributions of traits. For every pair of species, we removed traits that did not sum up to 50 counts. We also excluded GMM traits for central metabolism such as glycolysis and the pentose phosphate pathway among others and used only those associated with degradation, consumption or production. A list of GMMs and classification of trophic levels in provided in the Supplementary Table S2.

## Supplementary information

Supplementary Table S1

Supplementary Table S2

Supplementary Table S3

Supplementary Table S4

Supplementary Table S5

Supplementary Figures

## Data Availability

All necessary information to reproduce the analysis and figures is available at the GitHub repository (https://github.com/mibwurrepo/Shetty_et_al_MDbMM16) and Supplementary Notes. Metabolites data are available here https://github.com/mibwurrepo/Shetty_et_al_MDbMM16/blob/master/data/metabolites_hplc_mdbmm.csv. Custom R functions used for analysis and generating figures are available as a research compendium R package, *syncomR* (https://github.com/microsud/syncomR). The raw 16S rRNA amplicon sequencing and metatranscriptomics data are available at ENA under the study accession number PRJEB46578.
