# Supplementary material for: Dynamic metabolic interactions and trophic roles of human gut microbes identified using a minimal microbiome exhibiting ecological properties
Source: ISME J. 2022 Jun 18;16(9):2144–59. doi: 10.1038/s41396-022-01255-2 (PMC9381525; doi:10.1038/s41396-022-01255-2)
Supplement: Supplementary file 1 — Supplementary Table S1 [file 41396_2022_1255_MOESM1_ESM.docx]

**Supplementary Table S1: Prevalence of core species identified in 1155 gut metagenomes with a prevalence of >50% and minimum abundance threshold of 0.001%.** The 15 core species part of MDb-MM are highlighted in bold. The 16^th^ non-core species is *Blautia hydrogenotrophica.*

| Taxa | Prevalence (%) |
| --- | --- |
| ***Subdoligranulum unclassified*** | **99.83** |
| *Ruminococcus torques* | 99.74 |
| ***Faecalibacterium prausnitzii*** | **99.22** |
| ***Ruminococcus obeum*** | **98.27** |
| ***Eubacterium rectale*** | **97.14** |
| *Oscillibacter unclassified* | 95.58 |
| *Bacteroides uniformis* | 94.20 |
| *Dorea formicigenerans* | 92.55 |
| ***Eubacterium hallii*** | **91.77** |
| *Dorea longicatena* | 91.69 |
| ***Bacteroides ovatus*** | **90.82** |
| *Bacteroides vulgatus* | 90.82 |
| *Coprococcus comes* | 90.22 |
| *Roseburia inulinivorans* | 89.61 |
| *Roseburia hominis* | 89.35 |
| ***Lachnospiraceae bacterium 7 1 58FAA*** | **88.40** |
| *Lachnospiraceae bacterium 5 1 63FAA* | 87.19 |
| ***Collinsella aerofaciens*** | **85.89** |
| *Bifidobacterium longum* | 85.89 |
| *Alistipes putredinis* | 83.72 |
| *Clostridium leptum* | 83.46 |
| ***Coprococcus catus*** | **83.12** |
| *Anaerostipes hadrus* | 83.03 |
| *Alistipes shahii* | 82.42 |
| *Eubacterium eligens* | 82.25 |
| *Alistipes onderdonkii* | 82.25 |
| *Streptococcus salivarius* | 82.16 |
| *Bilophila unclassified* | 82.08 |
| ***Ruminococcus bromii*** | **82.08** |
| *Eubacterium ramulus* | 81.90 |
| *Bacteroides dorei* | 80.17 |
| *Lachnospiraceae bacterium 3 1 46FAA* | 79.91 |
| *Bacteroides thetaiotaomicron* | 78.53 |
| ***Bifidobacterium adolescentis*** | **78.01** |
| *Alistipes finegoldii* | 77.58 |
| *Barnesiella intestinihominis* | 77.58 |
| *Ruminococcus sp 5 1 39BFAA* | 77.14 |
| *Bacteroidales bacterium ph8* | 76.28 |
| *Eubacterium ventriosum* | 75.67 |
| ***Roseburia intestinalis*** | **75.24** |
| ***Bacteroides xylanisolvens*** | **74.63** |
| *Bacteroides caccae* | 73.42 |
| *Streptococcus parasanguinis* | 73.42 |
| *Parabacteroides merdae* | 72.03 |
| *Parabacteroides distasonis* | 71.34 |
| *Adlercreutzia equolifaciens* | 70.48 |
| ***Eubacterium siraeum*** | **69.18** |
| *Veillonella unclassified* | 64.24 |
| ***Akkermansia muciniphila*** | **63.98** |
| *Odoribacter splanchnicus* | 63.29 |
| *Escherichia coli* | 63.12 |
| *Anaerotruncus unclassified* | 61.73 |
| *Alistipes senegalensis* | 61.21 |
| *Bilophila wadsworthia* | 60.52 |
| *Clostridium bartlettii* | 59.91 |
| *Holdemania filiformis* | 57.84 |
| *Alistipes indistinctus* | 57.32 |
| *Streptococcus thermophilus* | 57.32 |
| *Peptostreptococcaceae noname unclassified* | 57.06 |
| *Ruminococcus lactaris* | 54.29 |
| *Bacteroides cellulosilyticus* | 52.21 |
| *Ruminococcus gnavus* | 51.00 |
| *Bacteroides stercoris* | 50.65 |
| *Eggerthella unclassified* | 50.56 |
