# Supplementary material for: Dynamic metabolic interactions and trophic roles of human gut microbes identified using a minimal microbiome exhibiting ecological properties
Source: ISME J. 2022 Jun 18;16(9):2144–59. doi: 10.1038/s41396-022-01255-2 (PMC9381525; doi:10.1038/s41396-022-01255-2)
Supplement: Supplementary file 2 — Supplementary Table S2 [file 41396_2022_1255_MOESM2_ESM.docx]

| **Supplementary Table S2: Gut metabolic modules that were characterized as part of trophic guilds.** Note that the module id were modified form original publication to incorporate the curation done for this research. | | | | | |
| --- | --- | --- | --- | --- | --- |
| **module_id** | **module_name** | **category_1** | **trophic_included** | **trophic_level** | **trophic_guild** |
| MF0097 | homoacetogenesis | gas metabolism | yes | 4 | trophic_guild_4 |
| MF0098 | hydrogen metabolism | gas metabolism | yes | 4 | trophic_guild_4 |
| MF0099 | methanogenesis - methyl-coM | gas metabolism | yes | 4 | trophic_guild_4 |
| MF0100 | methanogenesis from carbon dioxide | gas metabolism | yes | 4 | trophic_guild_4 |
| MF0101 | nitrate reduction (dissimilatory) | inorganic nutrient metabolism | yes | 4 | trophic_guild_4 |
| MF0102 | sulfate reduction (dissimilatory) | gas metabolism | yes | 4 | trophic_guild_4 |
| MF0059 | anaerobic fatty acid beta-oxidation | lipid degradation | yes | 3 | trophic_guild_3 |
| MF0075 | acetate to acetyl-CoA | organic acid metabolism | yes | 3 | trophic_guild_3 |
| MF0077 | formate conversion | organic acid metabolism | yes | 3 | trophic_guild_3 |
| MF0079 | lactate consumption I | organic acid metabolism | yes | 3 | trophic_guild_3 |
| MF0080 | lactate consumption II | organic acid metabolism | yes | 3 | trophic_guild_3 |
| MF0083 | succinate consumption | organic acid metabolism | yes | 3 | trophic_guild_3 |
| MF0104 | lactate consumption III (lctABCDEF, Curated) | organic acid metabolism | yes | 3 | trophic_guild_3 |
| MF0108 | propionate production IV (1,2-PD) | organic acid metabolism | yes | 3 | trophic_guild_3 |
| MF00075 | acetate to acetyl-CoA II | organic acid metabolism | yes | 3 | trophic_guild_3 |
| MF0006 | lactose degradation | carbohydrate degradation | yes | 2 | trophic_guild_2 |
| MF0007 | lactose and galactose degradation | carbohydrate degradation | yes | 2 | trophic_guild_2 |
| MF0008 | maltose degradation | carbohydrate degradation | yes | 2 | trophic_guild_2 |
| MF0009 | melibiose degradation | carbohydrate degradation | yes | 2 | trophic_guild_2 |
| MF0010 | sucrose degradation I | carbohydrate degradation | yes | 2 | trophic_guild_2 |
| MF0011 | sucrose degradation II | carbohydrate degradation | yes | 2 | trophic_guild_2 |
| MF0012 | trehalose degradation | carbohydrate degradation | yes | 2 | trophic_guild_2 |
| MF0013 | allose degradation | carbohydrate degradation | yes | 2 | trophic_guild_2 |
| MF0014 | arabinose degradation | carbohydrate degradation | yes | 2 | trophic_guild_2 |
| MF0015 | fructose degradation | carbohydrate degradation | yes | 2 | trophic_guild_2 |
| MF0016 | fucose degradation | carbohydrate degradation | yes | 2 | trophic_guild_2 |
| MF0017 | galactose degradation | carbohydrate degradation | yes | 2 | trophic_guild_2 |
| MF0018 | mannose degradation | carbohydrate degradation | yes | 2 | trophic_guild_2 |
| MF0019 | rhamnose degradation | carbohydrate degradation | yes | 2 | trophic_guild_2 |
| MF0020 | ribose degradation | carbohydrate degradation | yes | 2 | trophic_guild_2 |
| MF0021 | xylose degradation | carbohydrate degradation | yes | 2 | trophic_guild_2 |
| MF0022 | galacturonate degradation I | carbohydrate degradation | yes | 2 | trophic_guild_2 |
| MF0023 | galacturonate degradation II | carbohydrate degradation | yes | 2 | trophic_guild_2 |
| MF0060 | glycerol degradation I | lipid degradation | yes | 2 | trophic_guild_2 |
| MF0061 | glycerol degradation II | lipid degradation | yes | 2 | trophic_guild_2 |
| MF0062 | glycerol degradation III | lipid degradation | yes | 2 | trophic_guild_2 |
| MF0078 | lactaldehyde degradation | carbohydrate degradation | yes | 2 | trophic_guild_2 |
| MF0081 | methanol conversion | gas metabolism | yes | 2 | trophic_guild_2 |
| MF0105 | cellobiose degradation I (curated) | carbohydrate degradation | yes | 2 | trophic_guild_2 |
| MF0001 | arabinoxylan degradation | carbohydrate degradation | yes | 1 | trophic_guild_1 |
| MF0002 | fructan degradation (Curated) | carbohydrate degradation | yes | 1 | trophic_guild_1 |
| MF0003 | pectin degradation I | carbohydrate degradation | yes | 1 | trophic_guild_1 |
| MF0004 | pectin degradation II | carbohydrate degradation | yes | 1 | trophic_guild_1 |
| MF0005 | starch degradation | carbohydrate degradation | yes | 1 | trophic_guild_1 |
| MF0064 | triacylglycerol degradation | lipid degradation | yes | 1 | trophic_guild_1 |
| MF0103 | mucin degradation | glycoprotein degradation | yes | 1 | trophic_guild_1 |
| MF0024 | phenylalanine degradation | amino acid degradation | no | not_assigned | not_assigned |
| MF0025 | tryptophan degradation | amino acid degradation | no | not_assigned | not_assigned |
| MF0026 | tyrosine degradation I | amino acid degradation | no | not_assigned | not_assigned |
| MF0027 | tyrosine degradation II | amino acid degradation | no | not_assigned | not_assigned |
| MF0028 | aspartate degradation I | amino acid degradation | no | not_assigned | not_assigned |
| MF0029 | aspartate degradation II | amino acid degradation | no | not_assigned | not_assigned |
| MF0030 | glutamate degradation I | amino acid degradation | no | not_assigned | not_assigned |
| MF0031 | glutamate degradation II | amino acid degradation | no | not_assigned | not_assigned |
| MF0032 | glutamate degradation III | amino acid degradation | no | not_assigned | not_assigned |
| MF0033 | alanine degradation I | amino acid degradation | no | not_assigned | not_assigned |
| MF0034 | alanine degradation II | amino acid degradation | no | not_assigned | not_assigned |
| MF0035 | glycine degradation | amino acid degradation | no | not_assigned | not_assigned |
| MF0036 | isoleucine degradation | amino acid degradation | no | not_assigned | not_assigned |
| MF0037 | leucine degradation | amino acid degradation | no | not_assigned | not_assigned |
| MF0038 | methionine degradation I | amino acid degradation | no | not_assigned | not_assigned |
| MF0039 | methionine degradation II | amino acid degradation | no | not_assigned | not_assigned |
| MF0040 | proline degradation | amino acid degradation | no | not_assigned | not_assigned |
| MF0041 | valine degradation I | amino acid degradation | no | not_assigned | not_assigned |
| MF0042 | asparagine degradation | amino acid degradation | no | not_assigned | not_assigned |
| MF0043 | cysteine biosynthesis/homocysteine degradation | amino acid degradation | no | not_assigned | not_assigned |
| MF0044 | cysteine degradation I | amino acid degradation | no | not_assigned | not_assigned |
| MF0045 | cysteine degradation II | amino acid degradation | no | not_assigned | not_assigned |
| MF0046 | glutamine degradation I | amino acid degradation | no | not_assigned | not_assigned |
| MF0047 | glutamine degradation II | amino acid degradation | no | not_assigned | not_assigned |
| MF0048 | serine degradation | amino acid degradation | no | not_assigned | not_assigned |
| MF0049 | threonine degradation I | amino acid degradation | no | not_assigned | not_assigned |
| MF0050 | threonine degradation II | amino acid degradation | no | not_assigned | not_assigned |
| MF0051 | arginine degradation I | amino acid degradation | no | not_assigned | not_assigned |
| MF0052 | arginine degradation II | amino acid degradation | no | not_assigned | not_assigned |
| MF0053 | arginine degradation III | amino acid degradation | no | not_assigned | not_assigned |
| MF0054 | arginine degradation IV | amino acid degradation | no | not_assigned | not_assigned |
| MF0055 | arginine degradation V | amino acid degradation | no | not_assigned | not_assigned |
| MF0056 | histidine degradation | amino acid degradation | no | not_assigned | not_assigned |
| MF0057 | lysine degradation I | amino acid degradation | no | not_assigned | not_assigned |
| MF0058 | lysine degradation II | amino acid degradation | no | not_assigned | not_assigned |
| MF0063 | glyoxylate bypass | lipid degradation | no | not_assigned | not_assigned |
| MF0065 | Bifidobacterium shunt | central metabolism | no | not_assigned | not_assigned |
| MF0066 | Entner-Doudoroff pathway | central metabolism | no | not_assigned | not_assigned |
| MF0067 | glycolysis (preparatory phase) | central metabolism | no | not_assigned | not_assigned |
| MF0068 | glycolysis (pay-off phase) | central metabolism | no | not_assigned | not_assigned |
| MF0069 | NADH:ferredoxin oxidoreductase | gas metabolism | no | not_assigned | not_assigned |
| MF0070 | pentose phosphate pathway (oxidative phase) | central metabolism | no | not_assigned | not_assigned |
| MF0071 | pentose phosphate pathway (non-oxidative branch) | central metabolism | no | not_assigned | not_assigned |
| MF0072 | pyruvate dehydrogenase complex | central metabolism | no | not_assigned | not_assigned |
| MF0073 | pyruvate:ferredoxin oxidoreductase | central metabolism | no | not_assigned | not_assigned |
| MF0074 | pyruvate:formate lyase | central metabolism | no | not_assigned | not_assigned |
| MF0076 | 4-aminobutyrate degradation | amino acid degradation | no | not_assigned | not_assigned |
| MF0082 | putrescine degradation | amines and polyamines degradation | no | not_assigned | not_assigned |
| MF0084 | succinate conversion to propionate | organic acid metabolism | yes | not_assigned | not_assigned |
| MF0085 | urea degradation | amines and polyamines degradation | yes | not_assigned | not_assigned |
| MF0086 | acetyl-CoA to acetate | organic acid metabolism | no | not_assigned | not_assigned |
| MF0087 | acetyl-CoA to crotonyl-CoA | organic acid metabolism | no | not_assigned | not_assigned |
| MF0088 | butyrate production I | organic acid metabolism | yes | not_assigned | not_assigned |
| MF0089 | butyrate production II | organic acid metabolism | yes | not_assigned | not_assigned |
| MF0090 | ethanol production I | alcohol metabolism | yes | not_assigned | not_assigned |
| MF0091 | ethanol production II | alcohol metabolism | yes | not_assigned | not_assigned |
| MF0092 | lactate production | organic acid metabolism | yes | not_assigned | not_assigned |
| MF0093 | propionate production I | organic acid metabolism | yes | not_assigned | not_assigned |
| MF0094 | propionate production II | organic acid metabolism | yes | not_assigned | not_assigned |
| MF0095 | propionate production III | organic acid metabolism | yes | not_assigned | not_assigned |
| MF0096 | succinate production | organic acid metabolism | yes | not_assigned | not_assigned |
| MF0106 | 1,2 propanediol production I (lactaldehyde, curated) | diol metabolism | no | not_assigned | not_assigned |
| MF0107 | 1,2 propanediol production II (Hydroxyacetone, curated) | diol metabolism | no | not_assigned | not_assigned |
| MF00020 | serine biosynthesis, glycerate-3P => serine | amino acid biosynthesis | no | not_assigned | not_assigned |
| MF00018 | threonine biosynthesis, aspartate => homoserine => threonine | amino acid biosynthesis | no | not_assigned | not_assigned |
| MF00033 | ectoine biosynthesis, aspartate => ectoine | amino acid degradation | no | not_assigned | not_assigned |
| MF00021 | cysteine biosynthesis, serine => cysteine | amino acid biosynthesis | no | not_assigned | not_assigned |
| MF00338 | cysteine biosynthesis, homocysteine + serine => cysteine | amino acid biosynthesis | no | not_assigned | not_assigned |
| MF00609 | cysteine biosynthesis, methionine => cysteine | amino acid biosynthesis | no | not_assigned | not_assigned |
| MF00017 | methionine biosynthesis, apartate => homoserine => methionine | amino acid biosynthesis | no | not_assigned | not_assigned |
| MF00019 | valine/isoleucine biosynthesis, pyruvate => valine / 2-oxobutanoate => isoleucine | amino acid biosynthesis | no | not_assigned | not_assigned |
| MF00535 | isoleucine biosynthesis, pyruvate => 2-oxobutanoate | amino acid biosynthesis | no | not_assigned | not_assigned |
| MF00570 | isoleucine biosynthesis, threonine => 2-oxobutanoate => isoleucine | amino acid biosynthesis | no | not_assigned | not_assigned |
| MF00432 | leucine biosynthesis, 2-oxoisovalerate => 2-oxoisocaproate | amino acid biosynthesis | no | not_assigned | not_assigned |
| MF00016 | lysine biosynthesis, succinyl-DAP pathway, aspartate => lysine | amino acid biosynthesis | no | not_assigned | not_assigned |
| MF00525 | lysine biosynthesis, acetyl-DAP pathway, aspartate => lysine | amino acid biosynthesis | no | not_assigned | not_assigned |
| MF00526 | lysine biosynthesis, DAP dehydrogenase pathway, aspartate => lysine | amino acid biosynthesis | no | not_assigned | not_assigned |
| MF00527 | lysine biosynthesis, DAP aminotransferase pathway, aspartate => lysine | amino acid biosynthesis | no | not_assigned | not_assigned |
| MF00030 | lysine biosynthesis, AAA pathway, 2-oxoglutarate => 2-aminoadipate => lysine | amino acid biosynthesis | no | not_assigned | not_assigned |
| MF00433 | lysine biosynthesis, 2-oxoglutarate => 2-oxoadipate | amino acid biosynthesis | no | not_assigned | not_assigned |
| MF00031 | lysine biosynthesis, mediated by LysW, 2-aminoadipate => lysine | amino acid biosynthesis | no | not_assigned | not_assigned |
| MF00028 | ornithine biosynthesis, glutamate => ornithine | amino acid biosynthesis | no | not_assigned | not_assigned |
| MF00763 | ornithine biosynthesis, mediated by LysW, glutamate => ornithine | amino acid biosynthesis | no | not_assigned | not_assigned |
| MF00844 | arginine biosynthesis, ornithine => arginine | amino acid biosynthesis | no | not_assigned | not_assigned |
| MF00845 | arginine biosynthesis, glutamate => acetylcitrulline => arginine | amino acid biosynthesis | no | not_assigned | not_assigned |
| MF00029 | urea cycle | amines and polyamines degradation | no | not_assigned | not_assigned |
| MF00015 | proline biosynthesis, glutamate => proline | amino acid biosynthesis | no | not_assigned | not_assigned |
| MF00026 | histidine biosynthesis, PRPP => histidine | amino acid biosynthesis | no | not_assigned | not_assigned |
| MF00022 | shikimate pathway, phosphoenolpyruvate + erythrose-4P => chorismate | amino acid biosynthesis | no | not_assigned | not_assigned |
| MF00023 | tryptophan biosynthesis, chorismate => tryptophan | amino acid biosynthesis | no | not_assigned | not_assigned |
| MF00024 | phenylalanine biosynthesis, chorismate => phenylalanine | amino acid biosynthesis | no | not_assigned | not_assigned |
| MF00025 | tyrosine biosynthesis, chorismate => tyrosine | amino acid biosynthesis | no | not_assigned | not_assigned |
| MF00040 | tyrosine biosynthesis, prephanate => pretyrosine => tyrosine | amino acid biosynthesis | no | not_assigned | not_assigned |
| MGB006 | glutamate biosynthesis I | amino acid biosynthesis | no | not_assigned | not_assigned |
| MGB007 | glutamate biosynthesis II | amino acid biosynthesis | no | not_assigned | not_assigned |
| MGB044 | acetate synthesis II | organic acid metabolism | no | not_assigned | not_assigned |
| MGB045 | acetate synthesis III | organic acid metabolism | no | not_assigned | not_assigned |
| MGB046 | acetate synthesis IV | organic acid metabolism | no | not_assigned | not_assigned |
