# Supplementary material for: Dynamic metabolic interactions and trophic roles of human gut microbes identified using a minimal microbiome exhibiting ecological properties
Source: ISME J. 2022 Jun 18;16(9):2144–59. doi: 10.1038/s41396-022-01255-2 (PMC9381525; doi:10.1038/s41396-022-01255-2)
Supplement: Supplementary file 3 — Supplementary Table S3 [file 41396_2022_1255_MOESM3_ESM.docx]

| **Supplementary Table S3: Candidate strains used in this study.** All strains, except *C. catus* were grown for 24 h. *C. catus* was grown for 48 h due to its flow growth and to achieve sufficient biomass for the experiment. | | | | |
| --- | --- | --- | --- | --- |
| **Species** | **Strain** | **Inoculation (%)** | **16S rRNA gene copies/genome** | **Substrate pre-culture** |
| *A. muciniphila* | ATCC BAA-835 | 1 | 3 | Crude mucin |
| *B. ovatus* | HMP strain 3_8_47FAA | 1 | 2 | 20 mM galactose, 20 mM xylose and 0.2% starch |
| *B. xylanisolvens* | HMP strain 2_1_22 | 1 | 1 | 20 mM galactose, 20 mM xylose and 0.2% starch |
| *A. soehngenii* | DSM 17630 (L2-7) | 2 | 8 | 60 mM glucose |
| *C. catus* | ATCC 27761 | 1 | 1 | 60 mM glucose |
| *Flavonifractor plautii* | HMP strain 7_1_58FAA | 2 | 1 | 20 mM glucose, 20 mM galactose and 20 mM xylose |
| *E. sireaum* | DSM 15702 | 2 | 1 | 30 mM glucose, 20 mM maltose and 0.1% starch |
| *A. rectalis* | DSM 17629 | 1 | 5 | 20 mM glucose, 20 mM lactose and 20 mM xylose |
| *R. intestinalis* | DSM 14610 | 2 | 3 | 30 mM glucose, 20 mM maltose and 0.1% starch |
| *F. prausnitzii* | A2-165 | 1 | 2 | 30 mM glucose and 30 mM fructose |
| *S. variabile* | DSM 15176 | 1 | 4 | 60 mM glucose |
| *R. bromii* | ATCC 27255 | 2 | 2 | 30 mM glucose and 30 mM fructose |
| *B. obeum* | DSM 25238 | 2 | 5 | 30 mM glucose and 30 mM fructose |
| *C. aerofaciens* | DSM 3979 | 1 | 6 | 30 mM glucose, 20 mM maltose and 0.1% starch |
| *B. adolescentis* | L2-32 | 1 | 4 | 20 mM glucose, 20 mM lactose and 20 mM xylose |
| *B. hydrogenotrophica* | DSM 10507 | 2 | 1 | 60 mM glucose |
