# Supplementary material for: Dynamic metabolic interactions and trophic roles of human gut microbes identified using a minimal microbiome exhibiting ecological properties
Source: ISME J. 2022 Jun 18;16(9):2144–59. doi: 10.1038/s41396-022-01255-2 (PMC9381525; doi:10.1038/s41396-022-01255-2)
Supplement: Supplementary file 4 — Supplementary Table S4 [file 41396_2022_1255_MOESM4_ESM.docx]

**Supplementary Table S4:** Amplicon sequencing raw data information and total 16S rRNA gene copies in each sample. The last two columns contain percent of reads assigned to MDb-MM reads and not classified as MDb-MM strains.

| **Timepoint** | **SampleID** | **Raw reads** | **Filtered reads** | **Percent surviving** | **qPCR total 16S rRNA copies** | **MDb-MM (%)** | **Non-MDb-MM (%)** |
| --- | --- | --- | --- | --- | --- | --- | --- |
| 0 | F5T0 | 210840 | 206942 | 98.2 | 3811346.4 | 88.66 | 11.34 |
|  | F6T0 | 210157 | 206286 | 98.2 | 3486041.6 | 90.33 | 9.67 |
|  | F8T0 | 267888 | 262586 | 98.0 | 2576053.0 | 91.27 | 8.73 |
| 4 | F5T4 | 289071 | 282481 | 97.7 | 27489889.8 | 95.17 | 4.83 |
|  | F6T4 | 256351 | 252155 | 98.4 | 11919416.9 | 93.75 | 6.25 |
|  | F8T4 | 337504 | 331433 | 98.2 | 87488576.8 | 95.93 | 4.07 |
| 8 | F5T8 | 422420 | 414086 | 98.0 | 268629666.3 | 98.47 | 1.53 |
|  | F6T8 | 384591 | 377621 | 98.2 | 212352319.4 | 98.36 | 1.64 |
|  | F8T8 | 329572 | 323392 | 98.1 | 239534469.8 | 98.40 | 1.60 |
| 12 | F5T12 | 181667 | 178044 | 98.0 | 936122605.8 | 97.75 | 2.25 |
|  | F6T12 | 270986 | 266133 | 98.2 | 443360166.2 | 97.70 | 2.30 |
|  | F8T12 | 263445 | 257749 | 97.8 | 922600757.1 | 98.33 | 1.67 |
| 24 | F5T24 | 288674 | 281794 | 97.6 | 222347670.3 | 97.85 | 2.15 |
|  | F6T24 | 257533 | 252976 | 98.2 | 292764928.7 | 96.79 | 3.21 |
|  | F8T24 | 292276 | 286756 | 98.1 | 604937439.2 | 98.14 | 1.86 |
| 28 | F5T28 | 179955 | 176292 | 98.0 | 171579937.3 | 97.36 | 2.64 |
|  | F6T28 | 283110 | 278143 | 98.2 | 81282242.7 | 97.15 | 2.85 |
|  | F8T28 | 293628 | 287326 | 97.9 | 216556050.2 | 97.78 | 2.22 |
| 32 | F5T32 | 431612 | 423642 | 98.2 | 300904450.0 | 98.02 | 1.98 |
|  | F6T32 | 432546 | 424692 | 98.2 | 379349453.1 | 97.70 | 2.30 |
|  | F8T32 | 329900 | 323795 | 98.1 | 278021559.3 | 97.81 | 2.19 |
| 48 | F5T48 | 286647 | 281747 | 98.3 | 415567781.5 | 98.06 | 1.94 |
|  | F6T48 | 286379 | 280766 | 98.0 | 176832265.7 | 98.23 | 1.77 |
|  | F8T48 | 301374 | 297383 | 98.7 | 206863233.5 | 98.40 | 1.60 |
| 52 | F5T52 | 233339 | 222394 | 95.3 | 900844321.6 | 98.15 | 1.85 |
|  | F6T52 | 310861 | 306146 | 98.5 | 553743176.3 | 97.28 | 2.72 |
|  | F8T52 | 272200 | 266928 | 98.1 | 758584484.5 | 97.22 | 2.78 |
| 56 | F5T56 | 162171 | 159720 | 98.5 | 224283973.6 | 97.02 | 2.98 |
|  | F6T56 | 322208 | 315806 | 98.0 | 112548896.5 | 97.55 | 2.45 |
|  | F8T56 | 298463 | 293128 | 98.2 | 57974429.2 | 98.32 | 1.68 |
| 72 | F5T72 | 193876 | 190819 | 98.4 | 709672233.7 | 98.00 | 2.00 |
|  | F6T72 | 420939 | 412116 | 97.9 | 640409852.9 | 97.46 | 2.54 |
|  | F8T72 | 345906 | 339524 | 98.2 | 728797534.0 | 97.83 | 2.17 |
| 74 | F5T74 | 89989 | 88724 | 98.6 | 3340302331.0 | 98.48 | 1.52 |
|  | F6T74 | 212558 | 209228 | 98.4 | 347025789.6 | 99.15 | 0.85 |
|  | F8T74 | 245601 | 241253 | 98.2 | 331978131.1 | 99.31 | 0.69 |
| 76 | F5T76 | 230526 | 227019 | 98.5 | 111713348.3 | 97.66 | 2.34 |
|  | F6T76 | 257017 | 252626 | 98.3 | 102902522.9 | 98.00 | 2.00 |
|  | F8T76 | 327394 | 320770 | 98.0 | 98969912.5 | 97.92 | 2.08 |
| 80 | F5T80 | 248711 | 243642 | 98.0 | 539898202.1 | 97.75 | 2.25 |
|  | F6T80 | 382296 | 375316 | 98.2 | 999117561.6 | 97.87 | 2.13 |
|  | F8T80 | 276439 | 269318 | 97.4 | 178381303.3 | 97.91 | 2.09 |
| 80 | F5T80 | 347580 | 341800 | 98.3 | 156912853.6 | 97.90 | 2.10 |
|  | F6T80 | 112418 | 110163 | 98.0 | 212274142.0 | 98.30 | 1.70 |
|  | F8T80 | 294291 | 290517 | 98.7 | 422869144.3 | 98.01 | 1.99 |
| 96 | F5T96 | 365868 | 359464 | 98.2 | 19040516.2 | 98.13 | 1.87 |
|  | F6T96 | 388956 | 382045 | 98.2 | 168486235.1 | 98.39 | 1.61 |
|  | F8T96 | 471210 | 462344 | 98.1 | 82544366.4 | 98.25 | 1.75 |
| 100 | F5T100 | 408868 | 401376 | 98.2 | 191429422.6 | 97.20 | 2.80 |
|  | F6T100 | 294048 | 288605 | 98.1 | 476904931.3 | 98.09 | 1.91 |
|  | F8T100 | 338425 | 332501 | 98.2 | 233403880.2 | 98.48 | 1.52 |
| 104 | F5T104 | 137152 | 134527 | 98.1 | 390467572.3 | 97.62 | 2.38 |
|  | F6T104 | 39021 | 37355 | 95.7 | 451218769.7 | 97.87 | 2.13 |
|  | F8T104 | 131841 | 128709 | 97.6 | 601134955.6 | 97.47 | 2.53 |
| 120 | F5T120 | 338660 | 332616 | 98.2 | 196024068.3 | 97.97 | 2.03 |
|  | F6T120 | 228880 | 224573 | 98.1 | 586485314.9 | 97.45 | 2.55 |
|  | F8T120 | 163372 | 159914 | 97.9 | 477031944.8 | 97.75 | 2.25 |
| 124 | F5T124 | 153385 | 149463 | 97.4 | 95866766509.0 | 97.16 | 2.84 |
|  | F6T124 | 183696 | 180358 | 98.2 | 475763320.9 | 97.60 | 2.40 |
|  | F8T124 | 219202 | 214663 | 97.9 | 745523354.7 | 97.57 | 2.43 |
| 128 | F5T128 | 168687 | 165799 | 98.3 | 847714466.9 | 97.72 | 2.28 |
|  | F6T128 | 154708 | 151469 | 97.9 | 595189834.3 | 97.91 | 2.09 |
|  | F8T128 | 183450 | 179262 | 97.7 | 531853926.2 | 98.16 | 1.84 |
| 144 | F5T144 | 32695 | 31880 | 97.5 | 444916153.3 | 98.09 | 1.91 |
|  | F6T144 | 111683 | 109416 | 98.0 | 503724206.9 | 98.15 | 1.85 |
|  | F8T144 | 67768 | 66700 | 98.4 | 1463121363.0 | 97.81 | 2.19 |
| 148 | F5T148 | 377995 | 371462 | 98.3 | 151166193.0 | 98.05 | 1.95 |
|  | F6T148 | 388611 | 381639 | 98.2 | 788775873.3 | 98.28 | 1.72 |
|  | F8T148 | 488616 | 479462 | 98.1 | 212514528.5 | 98.24 | 1.76 |
| 152 | F5T152 | 79979 | 77608 | 97.0 | 243868402.6 | 97.83 | 2.17 |
|  | F6T152 | 237216 | 231590 | 97.6 | 347478949.8 | 98.79 | 1.21 |
|  | F8T152 | 214765 | 210479 | 98.0 | 355885846.5 | 98.92 | 1.08 |
| 168 | F5T168 | 112196 | 109419 | 97.5 | 420659464.2 | 99.36 | 0.64 |
|  | F6T168 | 89610 | 87863 | 98.1 | 330497181.9 | 97.85 | 2.15 |
|  | F8T168 | 136085 | 133557 | 98.1 | 615758408.8 | 97.87 | 2.13 |
| 172 | F5T172 | 75737 | 74460 | 98.3 | 266837025.0 | 97.41 | 2.59 |
|  | F6T172 | 177439 | 174079 | 98.1 | 556893816.9 | 97.96 | 2.04 |
|  | F8T172 | 162036 | 159218 | 98.3 | 272839232.3 | 97.49 | 2.51 |
| 173 | F5T173 | 161812 | 153651 | 95.0 | 156027000.5 | 97.75 | 2.25 |
|  | F6T173 | 176820 | 173056 | 97.9 | 330339381.5 | 97.42 | 2.58 |
|  | F8T173 | 213648 | 208980 | 97.8 | 225832216.3 | 97.62 | 2.38 |
| 176 | F5T176 | 210490 | 206388 | 98.1 | 80951607.6 | 97.82 | 2.18 |
|  | F6T176 | 167480 | 164131 | 98.0 | 85175651.0 | 97.89 | 2.11 |
|  | F8T176 | 169790 | 166588 | 98.1 | 78936721.3 | 97.66 | 2.34 |
| 192 | F5T192 | 242578 | 237194 | 97.8 | 137786044.5 | 98.12 | 1.88 |
|  | F6T192 | 75931 | 74582 | 98.2 | 207343758.1 | 97.18 | 2.82 |
|  | F8T192 | 85133 | 83860 | 98.5 | 94219228.2 | 97.56 | 2.44 |
| 196 | F5T196 | 421088 | 413078 | 98.1 | 545519592.3 | 98.06 | 1.94 |
|  | F6T196 | 271859 | 266700 | 98.1 | 873706311.7 | 98.15 | 1.85 |
|  | F8T196 | 312282 | 306701 | 98.2 | 860563021.5 | 97.59 | 2.41 |
| 200 | F5T200 | 179903 | 176895 | 98.3 | 342314548.2 | 97.76 | 2.24 |
|  | F6T200 | 173702 | 170053 | 97.9 | 224106034.3 | 97.84 | 2.16 |
|  | F8T200 | 166959 | 163260 | 97.8 | 330102734.6 | 97.98 | 2.02 |
| 216 | F5T216 | 202287 | 198344 | 98.1 | 360630081.9 | 97.81 | 2.19 |
|  | F6T216 | 149467 | 146145 | 97.8 | 348669878.0 | 98.19 | 1.81 |
|  | F8T216 | 198885 | 196021 | 98.6 | 322967882.2 | 98.05 | 1.95 |
| 220 | F5T220 | 340948 | 334363 | 98.1 | 171261464.5 | 97.95 | 2.05 |
|  | F6T220 | 429747 | 422259 | 98.3 | 291049825.1 | 97.89 | 2.11 |
|  | F8T220 | 303549 | 296468 | 97.7 | 198438613.7 | 98.45 | 1.55 |
| 224 | F5T224 | 290979 | 285129 | 98.0 | 352413111.5 | 97.92 | 2.08 |
|  | F6T224 | 287974 | 282895 | 98.2 | 193675021.2 | 97.52 | 2.48 |
|  | F8T224 | 238586 | 232434 | 97.4 | 355810051.0 | 98.38 | 1.62 |
| 240 | F5T240 | 233721 | 223697 | 95.7 | 215544316.7 | 98.49 | 1.51 |
|  | F6T240 | 285825 | 281579 | 98.5 | 384018604.1 | 98.08 | 1.92 |
|  | F8T240 | 346143 | 339661 | 98.1 | 233370625.0 | 98.36 | 1.64 |
| 244 | F5T244 | 106372 | 104144 | 97.9 | 1092769931.0 | 97.41 | 2.59 |
|  | F6T244 | 163055 | 160289 | 98.3 | 655598078.0 | 96.64 | 3.36 |
|  | F8T244 | 219925 | 216450 | 98.4 | 590764214.5 | 97.40 | 2.60 |
| 248 | F5T248 | 213043 | 209475 | 98.3 | 534199847.6 | 97.47 | 2.53 |
|  | F6T248 | 78381 | 75327 | 96.1 | 577591995.7 | 97.32 | 2.68 |
|  | F8T248 | 167425 | 164199 | 98.1 | 385333536.9 | 97.29 | 2.71 |
| 264 | F5T264 | 234603 | 229664 | 97.9 | 287490600.2 | 97.48 | 2.52 |
|  | F6T264 | 266624 | 261264 | 98.0 | 258908643.3 | 97.55 | 2.45 |
|  | F8T264 | 141639 | 137293 | 96.9 | 181813421.3 | 97.86 | 2.14 |
| 268 | F5T268 | 165188 | 162453 | 98.3 | 144846641.8 | 97.56 | 2.44 |
|  | F6T268 | 73166 | 70252 | 96.0 | 324617721.8 | 97.20 | 2.80 |
|  | F8T268 | 187429 | 183740 | 98.0 | 364339799.0 | 97.77 | 2.23 |
| 272 | F5T272 | 130408 | 127139 | 97.5 | 2462501607.0 | 97.74 | 2.26 |
|  | F6T272 | 369512 | 361808 | 97.9 | 764086186.9 | 97.71 | 2.29 |
|  | F8T272 | 348184 | 341568 | 98.1 | 1889205265.0 | 97.66 | 2.34 |
| 288 | F5T288 | 350815 | 343853 | 98.0 | 389926217.4 | 97.87 | 2.13 |
|  | F6T288 | 426277 | 418957 | 98.3 | 192033918.3 | 97.35 | 2.65 |
|  | F8T288 | 365267 | 356972 | 97.7 | 443261372.0 | 97.76 | 2.24 |
| 292 | F6T292 | 244145 | 239296 | 98.0 | 63050321.2 | 97.87 | 2.13 |
|  | F8T292 | 224190 | 219609 | 98.0 | 77310402.0 | 94.25 | 5.75 |
|  | F5T292 | 222344 | 218095 | 98.1 | 27582857.1 | 97.82 | 2.18 |
| 296 | F5T296 | 74723 | 73541 | 98.4 | 441374757.3 | 97.47 | 2.53 |
|  | F6T296 | 150069 | 147349 | 98.2 | 353585909.5 | 97.71 | 2.29 |
|  | F8T296 | 164958 | 162174 | 98.3 | 341824423.5 | 97.21 | 2.79 |
| 312 | F5T312 | 274438 | 268590 | 97.9 | 1210623309.0 | 97.92 | 2.08 |
|  | F6T312 | 443578 | 435392 | 98.2 | 245447356.5 | 99.06 | 0.94 |
|  | F8T312 | 403761 | 396856 | 98.3 | 375050587.9 | 98.00 | 2.00 |
| 316 | F5T316 | 191052 | 187028 | 97.9 | 818296324.6 | 98.45 | 1.55 |
|  | F6T316 | 70165 | 68929 | 98.2 | 669411208.0 | 97.92 | 2.08 |
|  | F8T316 | 98923 | 97413 | 98.5 | 542521525.7 | 97.35 | 2.65 |
| 320 | F5T320 | 337777 | 331944 | 98.3 | 783894145.6 | 97.81 | 2.19 |
|  | F6T320 | 208340 | 204445 | 98.1 | 440390676.9 | 97.10 | 2.90 |
|  | F8T320 | 136861 | 133960 | 97.9 | 715022138.2 | 97.74 | 2.26 |
| 336 | F5T336 | 263710 | 258282 | 97.9 | 216505506.3 | 97.15 | 2.85 |
|  | F6T336 | 268277 | 262252 | 97.8 | 583499608.2 | 96.79 | 3.21 |
|  | F8T336 | 261637 | 257292 | 98.3 | 354471362.9 | 96.88 | 3.12 |
| 340 | F5T340 | 51517 | 50393 | 97.8 | 750163444.0 | 97.54 | 2.46 |
|  | F6T340 | 161744 | 158917 | 98.3 | 754064898.6 | 98.22 | 1.78 |
|  | F8T340 | 93422 | 92077 | 98.6 | 588347287.7 | 97.39 | 2.61 |
| 344 | F5T344 | 214682 | 211447 | 98.5 | 36032437.0 | 97.60 | 2.40 |
|  | F6T344 | 213417 | 209475 | 98.2 | 591433996.6 | 97.46 | 2.54 |
|  | F8T344 | 303193 | 296988 | 98.0 | 85602362.6 | 97.27 | 2.73 |
| 365 | F5T365 | 249317 | 245911 | 98.6 | 1219663043.0 | 96.54 | 3.46 |
|  | F6T365 | 200982 | 197523 | 98.3 | 422532660.8 | 94.63 | 5.37 |
|  | F8T365 | 249676 | 245215 | 98.2 | 910852430.0 | 95.80 | 4.20 |
| 389 | F5T389 | 142083 | 138950 | 97.8 | 132931719.7 | 95.04 | 4.96 |
|  | F6T389 | 197856 | 193697 | 97.9 | 105111792.2 | 96.47 | 3.53 |
|  | F8T389 | 157593 | 153641 | 97.5 | 104901279.4 | 92.76 | 7.24 |
| 390 | F5T390 | 77908 | 76143 | 97.7 | 283078844.4 | 95.41 | 4.59 |
|  | F6T390 | 281946 | 276140 | 97.9 | 22730688.7 | 97.09 | 2.91 |
|  | F8T390 | 339244 | 332975 | 98.2 | 1295842650.0 | 93.46 | 6.54 |
| 408 | F5T408 | 174450 | 170261 | 97.6 | 268315303.8 | 92.88 | 7.12 |
|  | F6T408 | 190247 | 185582 | 97.5 | 270678668.8 | 96.19 | 3.81 |
|  | F8T408 | 163435 | 160150 | 98.0 | 207804613.0 | 92.10 | 7.90 |
| 412 | F5T412 | 103855 | 101676 | 97.9 | 929399946.5 | 92.83 | 7.17 |
|  | F6T412 | 176445 | 173402 | 98.3 | 676731413.4 | 97.89 | 2.11 |
|  | F8T412 | 198037 | 195009 | 98.5 | 708764184.1 | 93.90 | 6.10 |
| 416 | F5T416 | 278198 | 272688 | 98.0 | 988667622.9 | 94.35 | 5.65 |
|  | F6T416 | 288975 | 282533 | 97.8 | 1402823509.0 | 95.77 | 4.23 |
|  | F8T416 | 244715 | 240297 | 98.2 | 407296698.6 | 95.61 | 4.39 |
| 432 | F5T432 | 55443 | 54430 | 98.2 | 372236302.0 | 96.74 | 3.26 |
|  | F6T432 | 113149 | 110776 | 97.9 | 323119950.3 | 97.40 | 2.60 |
|  | F8T432 | 113635 | 111354 | 98.0 | 256142659.2 | 92.87 | 7.13 |
| 434 | F5T434 | 178198 | 173784 | 97.5 | 474662304.4 | 94.06 | 5.94 |
|  | F6T434 | 235014 | 230232 | 98.0 | 401200912.7 | 97.18 | 2.82 |
|  | F8T434 | 244691 | 239931 | 98.1 | 1050501826.0 | 93.91 | 6.09 |
| 436 | F5T436 | 227510 | 222919 | 98.0 | 959072907.8 | 97.54 | 2.46 |
|  | F6T436 | 164048 | 160612 | 97.9 | 660149532.3 | 94.33 | 5.67 |
|  | F8T436 | 95561 | 93271 | 97.6 | 346499010.0 | 94.34 | 5.66 |
| 438 | F5T438 | 113857 | 110825 | 97.3 | 379745452.1 | 95.02 | 4.98 |
|  | F6T438 | 41071 | 40268 | 98.0 | 634776777.4 | 96.75 | 3.25 |
|  | F8T438 | 53752 | 52849 | 98.3 | 417160091.3 | 94.76 | 5.24 |
| 440 | F5T440 | 214101 | 210367 | 98.3 | 129244967.9 | 94.70 | 5.30 |
|  | F6T440 | 236084 | 231795 | 98.2 | 359900591.5 | 96.73 | 3.27 |
|  | F8T440 | 148357 | 144614 | 97.5 | 106115248.7 | 95.04 | 4.96 |
| 456 | F5T456 | 262064 | 256565 | 97.9 | 563782664.3 | 91.45 | 8.55 |
|  | F6T456 | 392966 | 385848 | 98.2 | 1954787393.0 | 98.15 | 1.85 |
|  | F8T456 | 554165 | 544757 | 98.3 | 1854261984.0 | 91.59 | 8.41 |
| 460 | F5T460 | 67963 | 66529 | 97.9 | 1302747839.0 | 91.17 | 8.83 |
|  | F6T460 | 162087 | 159239 | 98.2 | 969977502.4 | 96.99 | 3.01 |
|  | F8T460 | 98527 | 97089 | 98.5 | 615081513.3 | 87.51 | 12.49 |
