# Supplementary material for: Dynamic metabolic interactions and trophic roles of human gut microbes identified using a minimal microbiome exhibiting ecological properties
Source: ISME J. 2022 Jun 18;16(9):2144–59. doi: 10.1038/s41396-022-01255-2 (PMC9381525; doi:10.1038/s41396-022-01255-2)
Supplement: Supplementary file 5 — Supplementary Table S5 [file 41396_2022_1255_MOESM5_ESM.docx]

| **Supplementary Table S5: Reads statistics for metatranscriptome sequencing.** | | | | | | | |
| --- | --- | --- | --- | --- | --- | --- | --- |
| **Timepoint** | **Sample ID** | **Raw** | **Clean** | **Error** | **Q20(%)** | **Q30(%)** | **GC** |
|  |  | **Reads** | **Reads** | **Rate (%)** |  |  | **Content (%)** |
| 24 h | F5T24 | 22510218 | 22345873 | 0.03 | 97.78 | 93.59 | 48.05 |
|  | F6T24 | 22505088 | 22301265 | 0.03 | 97.78 | 93.62 | 48.65 |
|  | F8T24 | 23938428 | 23653126 | 0.03 | 97.96 | 94.03 | 48.69 |
| 28 h | F5T28 | 23445687 | 23169642 | 0.03 | 97.77 | 93.53 | 48.47 |
|  | F6T28 | 20915651 | 20587416 | 0.03 | 97.66 | 93.36 | 48.02 |
|  | F8T28 | 31023722 | 30772505 | 0.03 | 97.66 | 93.34 | 47.64 |
| 32 h | F5T32 | 23196573 | 23016159 | 0.03 | 97.55 | 93.03 | 47.66 |
|  | F6T32 | 21059415 | 20866971 | 0.03 | 97.6 | 93.16 | 47.19 |
|  | F8T32 | 23431154 | 23235304 | 0.03 | 97.59 | 93.15 | 47.6 |
| 48 h | F5T48 | 24581426 | 24364344 | 0.03 | 97.09 | 92.07 | 50.8 |
|  | F6T48 | 27669986 | 27431356 | 0.03 | 97.75 | 93.59 | 49.73 |
|  | F8T48 | 19975946 | 19614457 | 0.03 | 97.88 | 93.89 | 49.71 |
| 52 h | F5T52 | 24154417 | 23852997 | 0.03 | 97.31 | 92.42 | 49.96 |
|  | F6T52 | 24977274 | 24693836 | 0.03 | 97.7 | 93.43 | 48.82 |
|  | F8T52 | 21691291 | 21492803 | 0.03 | 97.78 | 93.56 | 47.06 |
| 56 h | F5T56 | 24368167 | 24092192 | 0.03 | 97.78 | 93.59 | 48.86 |
|  | F6T56 | 21551870 | 21264878 | 0.03 | 97.93 | 93.98 | 49.3 |
|  | F8T56 | 20884681 | 20654025 | 0.03 | 97.71 | 93.45 | 47.25 |
| 74.5h | F5T74T5 | 24991237 | 24633215 | 0.03 | 97.37 | 92.79 | 53.47 |
|  | F6T74T5 | 24787587 | 24424072 | 0.03 | 97.8 | 93.8 | 49.12 |
|  | F8T74T5 | 21061858 | 20831029 | 0.03 | 97.83 | 93.72 | 47.99 |
| 152 h | F5T152 | 20126710 | 19789707 | 0.03 | 97 | 91.88 | 49.77 |
|  | F6T152 | 19982195 | 19754286 | 0.03 | 97.06 | 92 | 49.7 |
|  | F8T152 | 23065403 | 22876366 | 0.03 | 96.97 | 91.8 | 49.36 |
| 176 h | F5T176 | 25409034 | 25132415 | 0.03 | 97.39 | 92.63 | 49.14 |
|  | F6T176 | 23946432 | 23727878 | 0.03 | 96.97 | 91.73 | 49.08 |
|  | F8T176 | 20407642 | 20315381 | 0.03 | 97.12 | 92.12 | 49.61 |
| 240 h | F5T240 | 23439378 | 23121953 | 0.03 | 97.32 | 92.55 | 51.87 |
|  | F6T240 | 21489739 | 21337826 | 0.03 | 97.02 | 91.9 | 49.25 |
|  | F8T240 | 21267837 | 21029334 | 0.03 | 97.1 | 92.06 | 49.72 |
| 248 h | F5T248 | 21525048 | 21248891 | 0.03 | 96.94 | 91.72 | 49.27 |
|  | F6T248 | 24239734 | 23942665 | 0.03 | 97.16 | 92.14 | 47.8 |
|  | F8T248 | 24161528 | 23732221 | 0.03 | 97.3 | 92.52 | 49.27 |
| 264 h | F5T264 | 23474287 | 23110742 | 0.03 | 97.19 | 92.23 | 49.89 |
|  | F6T264 | 20955892 | 20733904 | 0.03 | 96.97 | 91.84 | 49.35 |
|  | F8T264 | 19904164 | 19601841 | 0.03 | 96.98 | 91.76 | 48.87 |
