# Supplementary material for: Dynamic metabolic interactions and trophic roles of human gut microbes identified using a minimal microbiome exhibiting ecological properties
Source: ISME J. 2022 Jun 18;16(9):2144–59. doi: 10.1038/s41396-022-01255-2 (PMC9381525; doi:10.1038/s41396-022-01255-2)
Supplement: Supplementary file 6 — Supplementary Figures [file 41396_2022_1255_MOESM6_ESM.docx]

**Supplementary Figures**

**Dynamic metabolic interactions and trophic roles of human gut microbes identified using a minimal microbiome exhibiting ecological properties**

Sudarshan A. Shetty ^1#⸸^*, Ioannis Kostopoulos ^1#⁋^, Sharon Y. Geerlings ^1#^, Hauke Smidt ^1^*, Willem M. de Vos ^1,2,^*, Clara Belzer ^1,^*

^1^Laboratory of Microbiology, Wageningen University & Research, Wageningen, The Netherlands

^2^Human Microbiome Research Program, Faculty of Medicine, University of Helsinki, Helsinki, Finland

^⸸^Present address: University Medical Center Groningen, Groningen, The Netherlands

^⁋^Present address: Danone Nutricia Research, Utrecht, The Netherlands

^#^These authors contributed equally

*Co-Corresponding authors

**Supplementary Data**


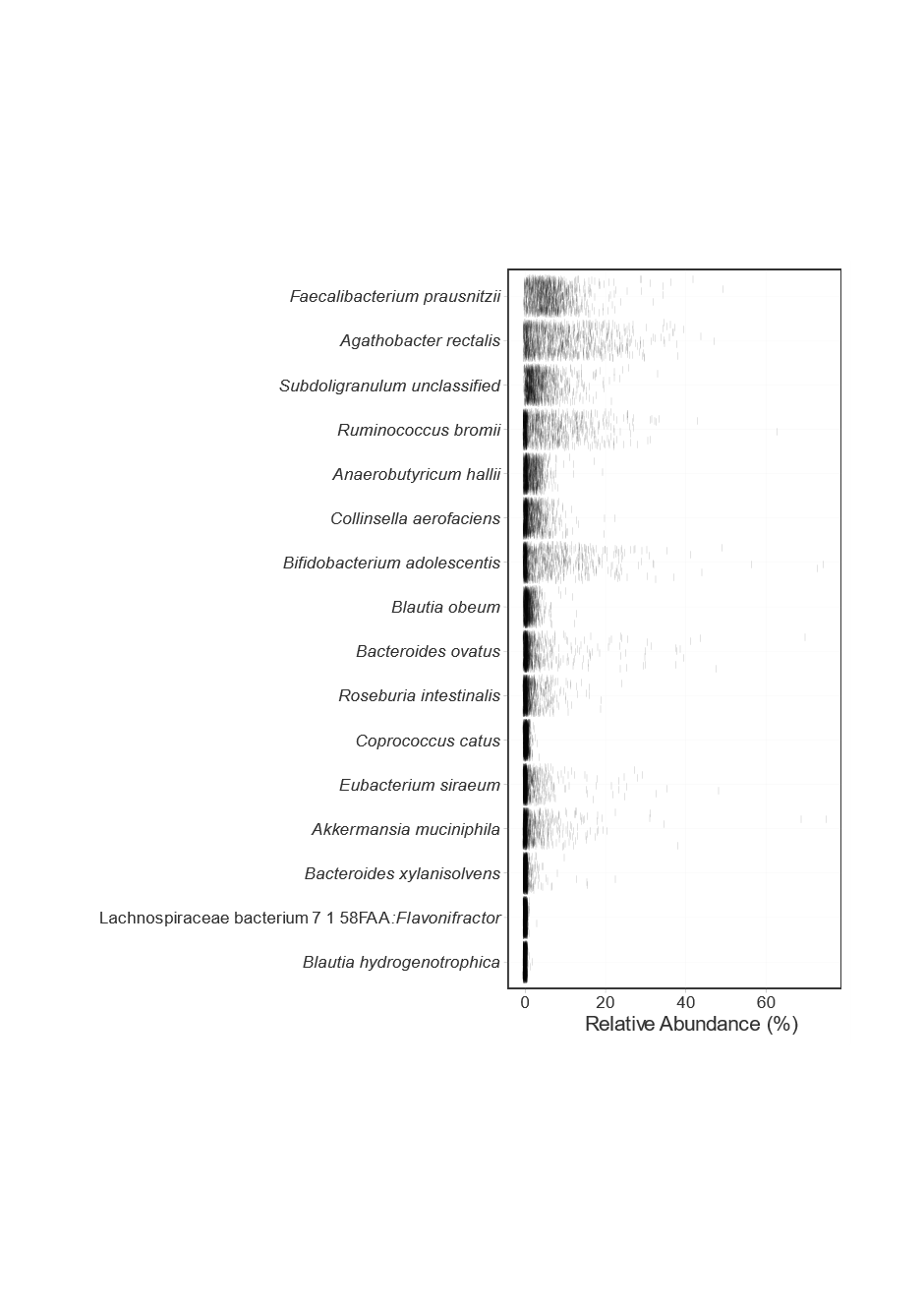


**Supplementary figure S1: Relative abundance of MDb-MM species in 1155 human gut metagenomes.** Each line represents one sample. Except for *B.hydrogenotrophica*, all other strains are part of the core microbiota at a relative abundance cut-off of 0.001% and prevalence of >50%.


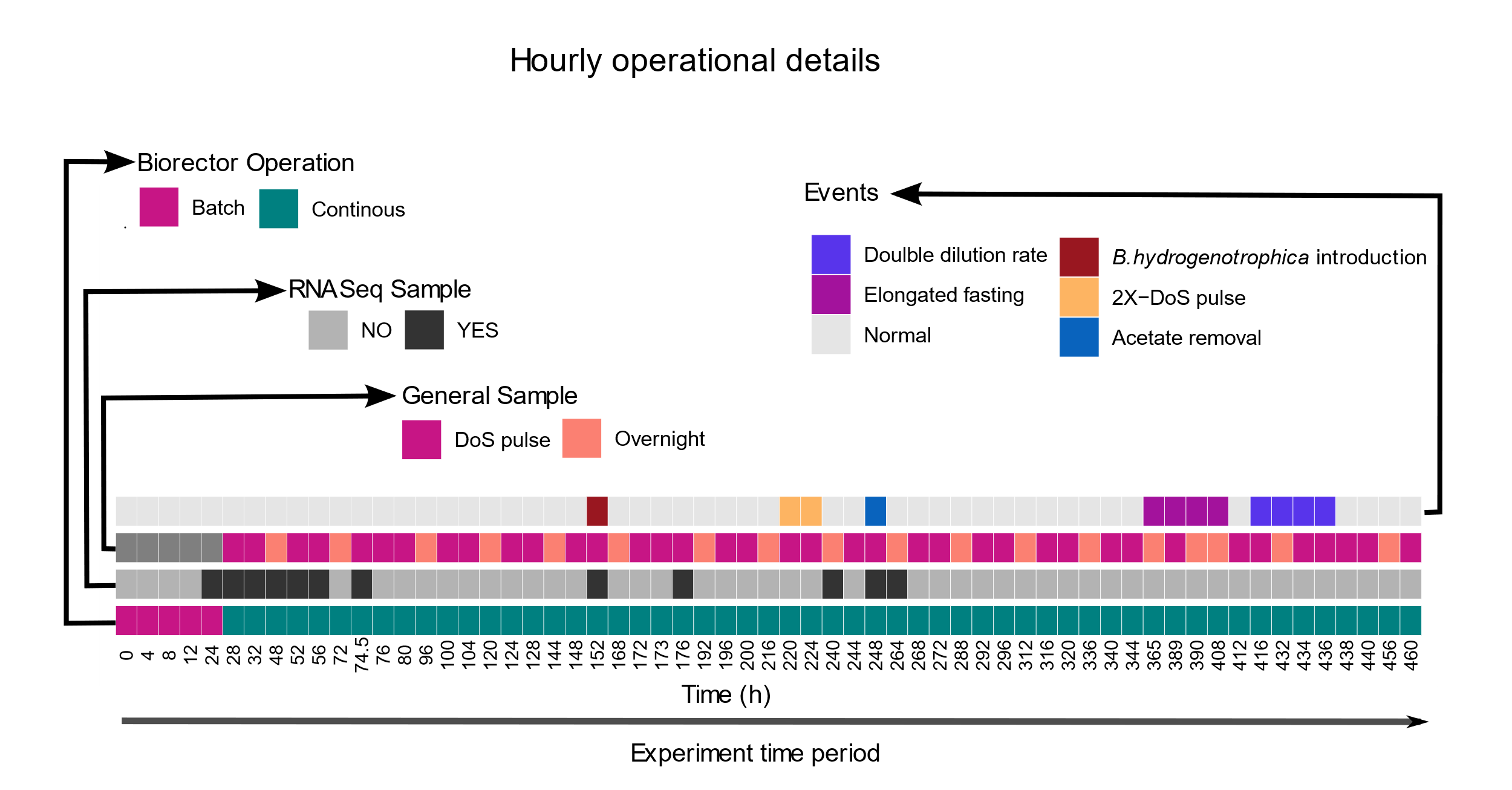


**Supplementary figure S2: Detailed sampling timepoints.** For all the time points shown here samples were collected for metabolites and 16S rRNA gene analysis. For RNASeq, 12 time points were chosen for analysis.


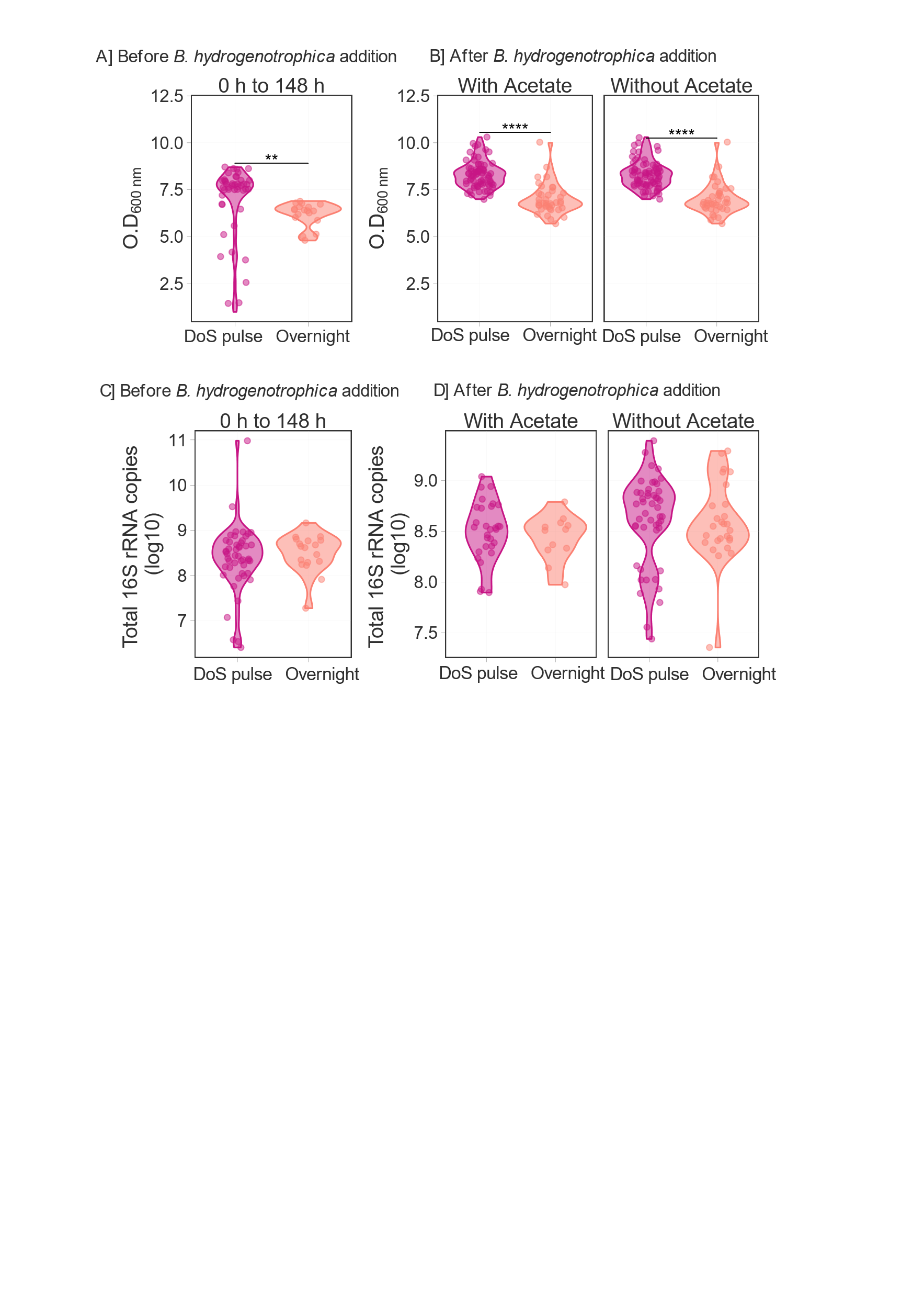


**Supplementary figure S3: Comparison of optical density and total 16S rRNA gene copies.** A) Comparison of optical density (O.D600) after DoS pulse (n= 48) and in overnight (n= 18) samples before addition of *B.hydrogenotrophica*  B) Comparison of optical density (O.D600) after DoS pulse and in overnight samples after addition of *B.hydrogenotrophica* with (DoS, n= 30, Overnight, n= 12) and without exogenous acetate (DoS, n= 48, Overnight, n= 27). C) Comparison of total 16S rRNA gene copies after DoS pulse (n= 48) and in overnight samples (n= 18) before addition of *B.hydrogenotrophica*  D) Comparison of total 16S rRNA gene copies after DoS pulse and in overnight samples after addition of *B.hydrogenotrophica* with acetate (DoS, n= 30, Overnight, n= 12) and without exogenous acetate (DoS, n= 48, Overnight, n= 27). The counts were transformed to log10. We used Wilcoxon test for pair-wise comparison corrected for multiple testing using the Benjamini–Hochberg FDR method. * = *p* < 0.05, ** = *p* < 0.001 and *** = *p* < 0.0001.


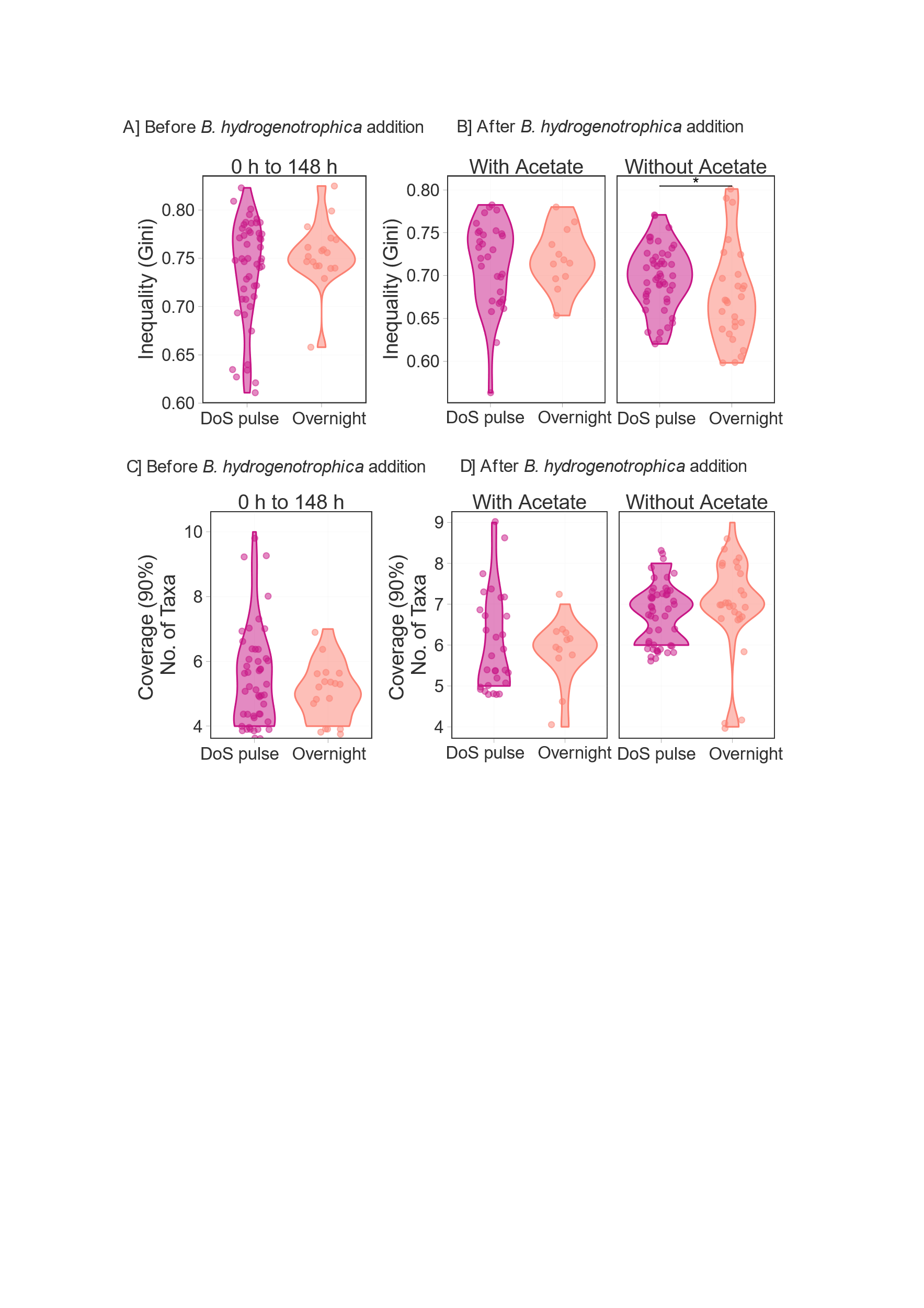


**Supplementary figure S4: Comparison of inequality and coverage.** A) Comparison of community evenness after DoS pulse (n= 48) and in overnight samples (n= 18) before addition of *B.hydrogenotrophica.* B) Comparison of community evenness after DoS pulse and in overnight samples after addition of *B.hydrogenotrophica* with acetate (DoS, n= 30, Overnight, n= 12) and without (DoS, n= 48, Overnight, n= 27) exogenous acetate. C) Comparison of total number of species contributing to 90% of the total abundance after DoS pulse and in overnight samples before addition of *B.hydrogenotrophica*  D) Comparison of total number of species contributing to 90% of the total abundance after DoS pulse (n= 48) and in overnight samples (n= 18) after addition of *B.hydrogenotrophica* with acetate (DoS, n= 30, Overnight, n= 12) and without (DoS, n= 48, Overnight, n= 27) exogenous acetate. We used Wilcoxon test for pair-wise comparison corrected for multiple testing using the Benjamini–Hochberg FDR method. * = *p* < 0.05, ** = *p* < 0.001 and *** = *p* < 0.0001.


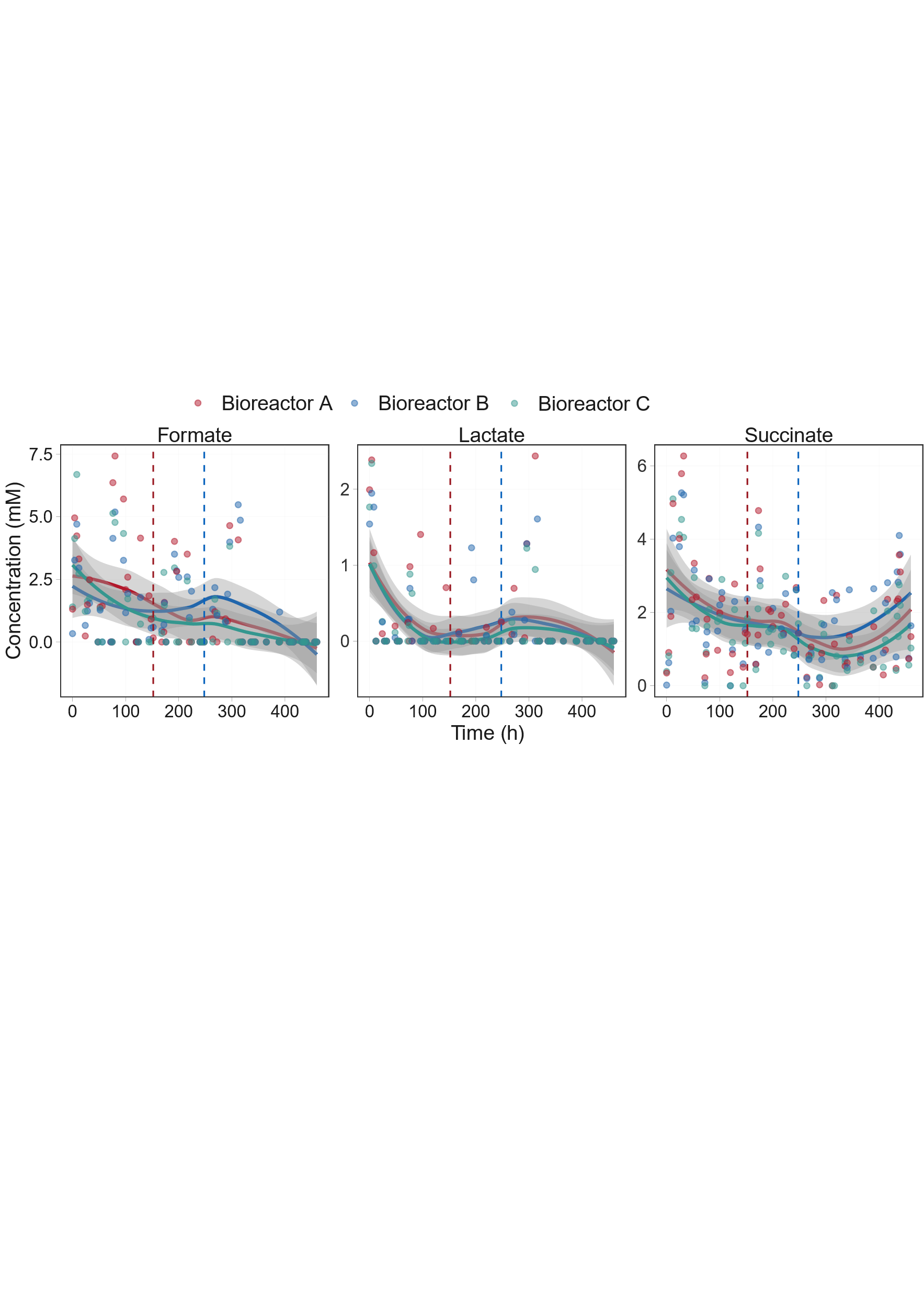


**Supplementary figure S5:** Concentration of SCFAs detected in minor concentration in the three bioreactors. The vertical dashed red lines indicates introduction of *B. hydrogenotrophica*(152 h) and blue line indicates removal of acetate/feed change (248h). ​The curved line represent the locally weighted smoothing (LOESS) for each of the bioreactors and the grey shaded region around these lines shows 95% confidence intervals for the fit. This was calculated and visualized with the default geom_smooth function in ggplot2 R package.


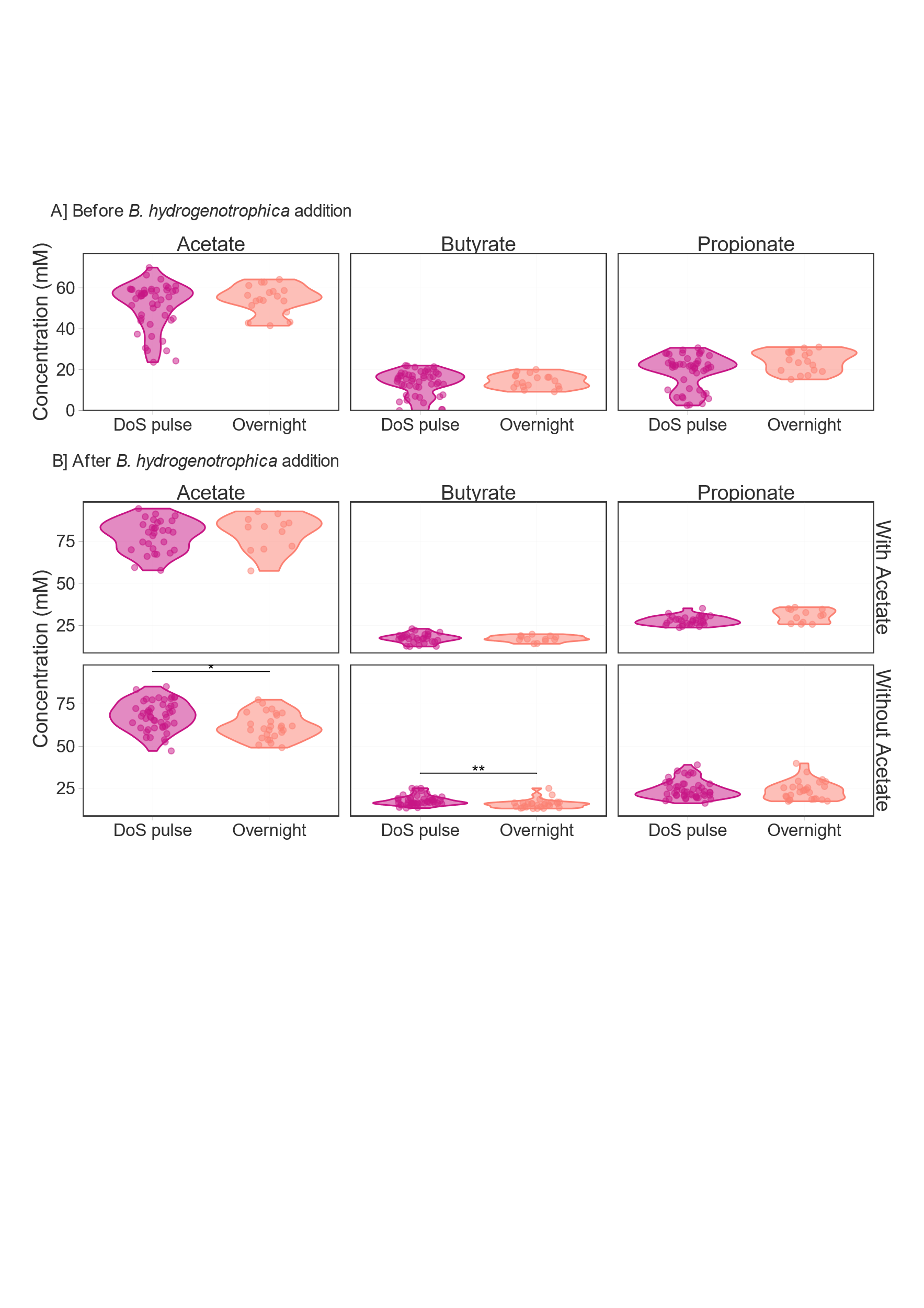


**Supplementary figure S6:** **Comparison of SCFA production.** A) The first 148 h before introduction of any disturbance event in the system (DoS pulse, n= 48 and Overnight, n= 18). B) After introduction of *B. hydrogenotrophica* as well as removal of acetate. With acetate number of samples for DoS pulse, n= 30 and for overnight, n= 12. Without acetate number of samples for DoS pulse, n= 48 and for overnight, n= 27. We used Wilcoxon test for pair-wise comparison corrected for multiple testing using the Benjamini–Hochberg FDR method. * = *p* < 0.05, ** = *p* < 0.001 and *** = *p* < 0.0001.


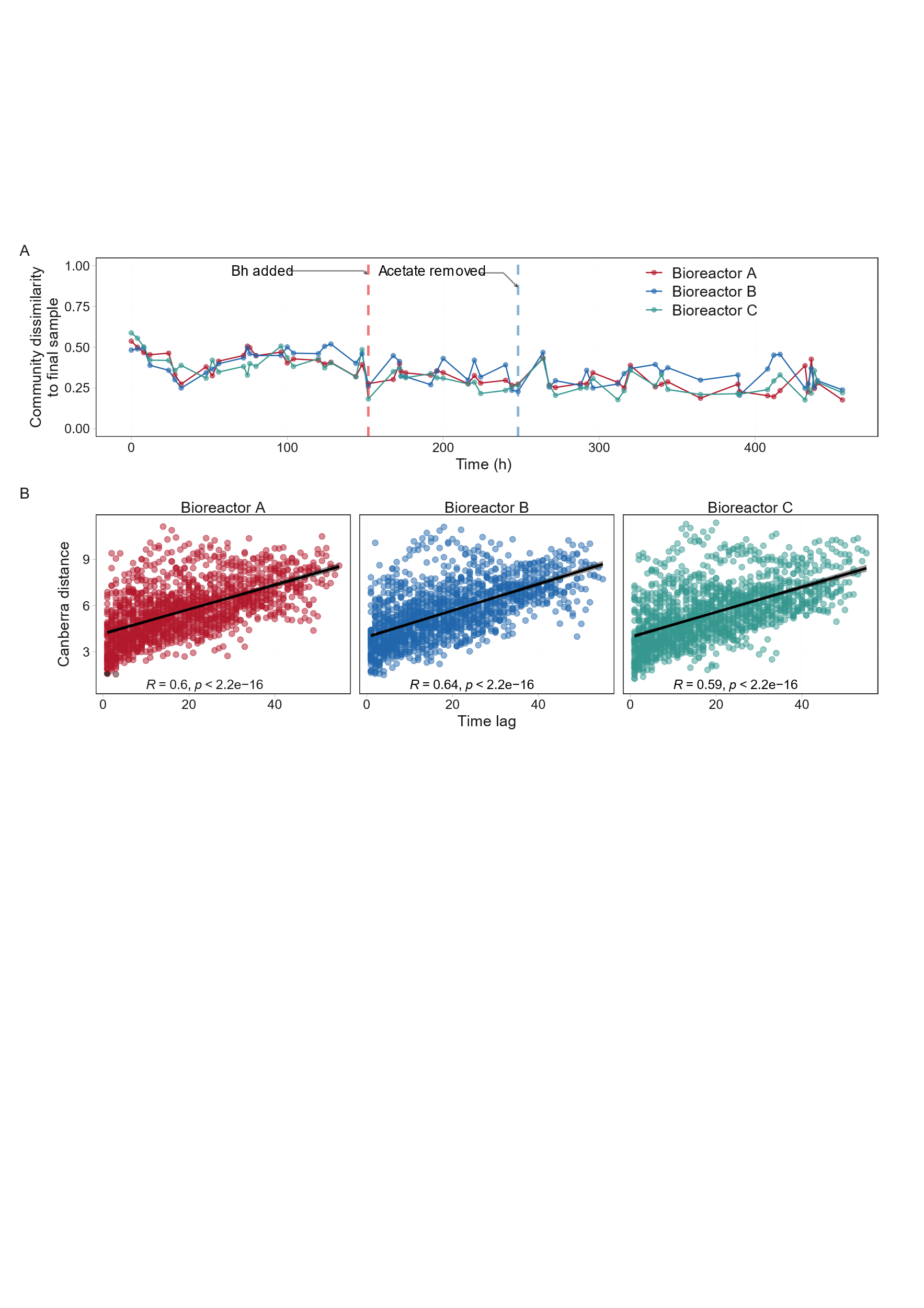


**Supplementary figure S7: Temporal patterns in community succession.** A) Temporal convergence patterns of MDb-MM in the three bioreactors (Canberra distance). B) Community change over time based on Canberra distances, with modified codes from the codyn R package which uses Euclidean distances.


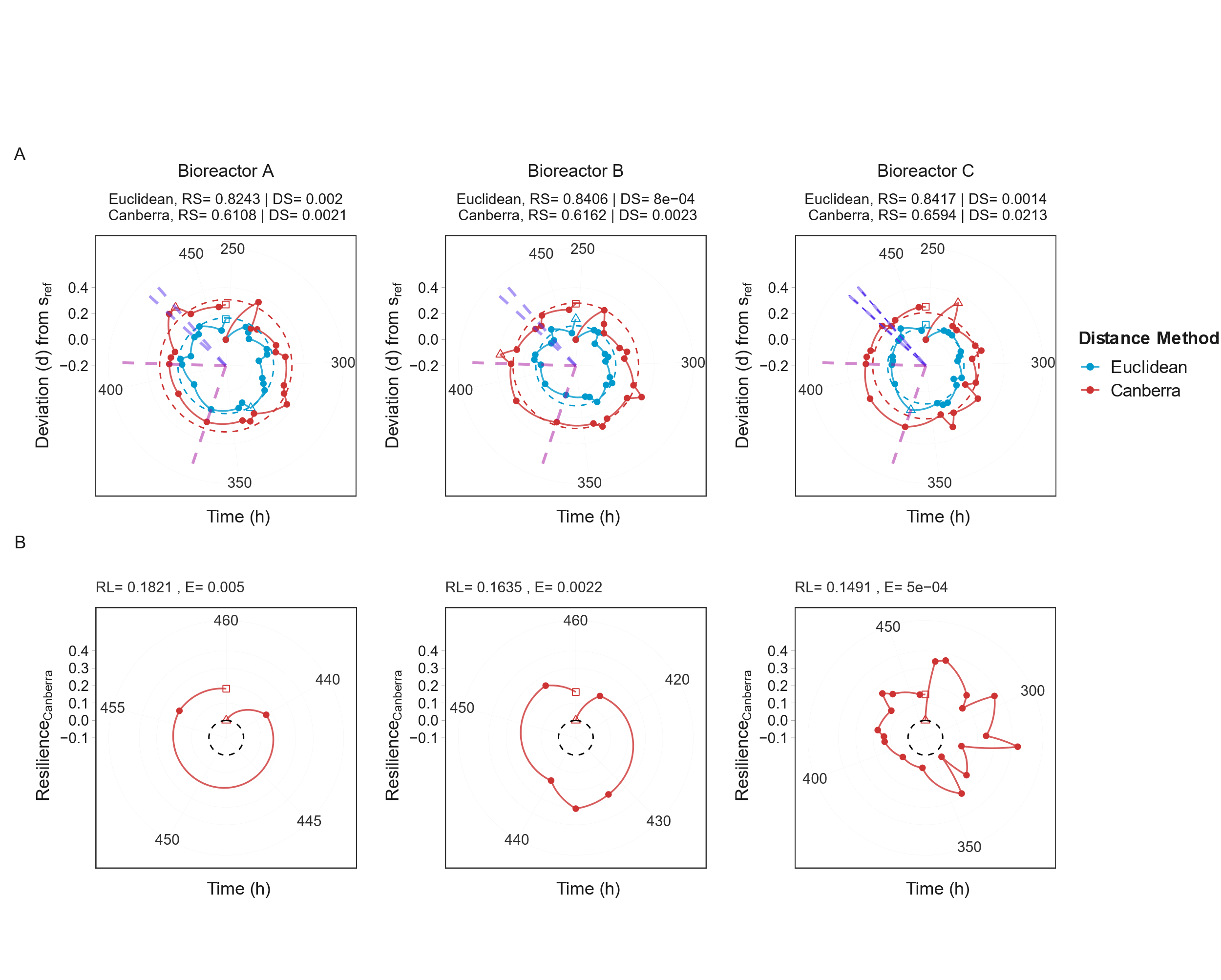


**Supplementary figure S8: Stability properties of MDb-MM in presence of multiple perturbations.** A) Community changes from the reference state calculated using Canberra and Euclidean distance. The reference boundary was calculated using the method described by Liu et al., 2018. The shaded region and brown dashed line depict reference boundary based on Canberra distance, while the blue dashed line depicts reference state boundary based on Euclidean distance. The hollow triangles represent timepoints when maximal deviation from reference state was observed. The lavender-coloured dashed line indicates elongated fasting samples and blue coloured dashed line indicates the doubling of dilution rate from 10ml/h to 20ml/h. B) Resilience of the MDb-MM in presence of multiple perturbations. The black dashed line depicts the reference boundary for deviation from disturbance event based on Canberra distance. The stability was calculated with 152 h (introduction of *B.hydrogenotrophica*) as the starting time, removal of acetate/feed change (248h) as the specific disturbance event and experiment end point was 460 h, when the experiment was ended. Abbreviations: RS, resistance; DS, displacement speed; RL, resilience and DS, displacement speed.​


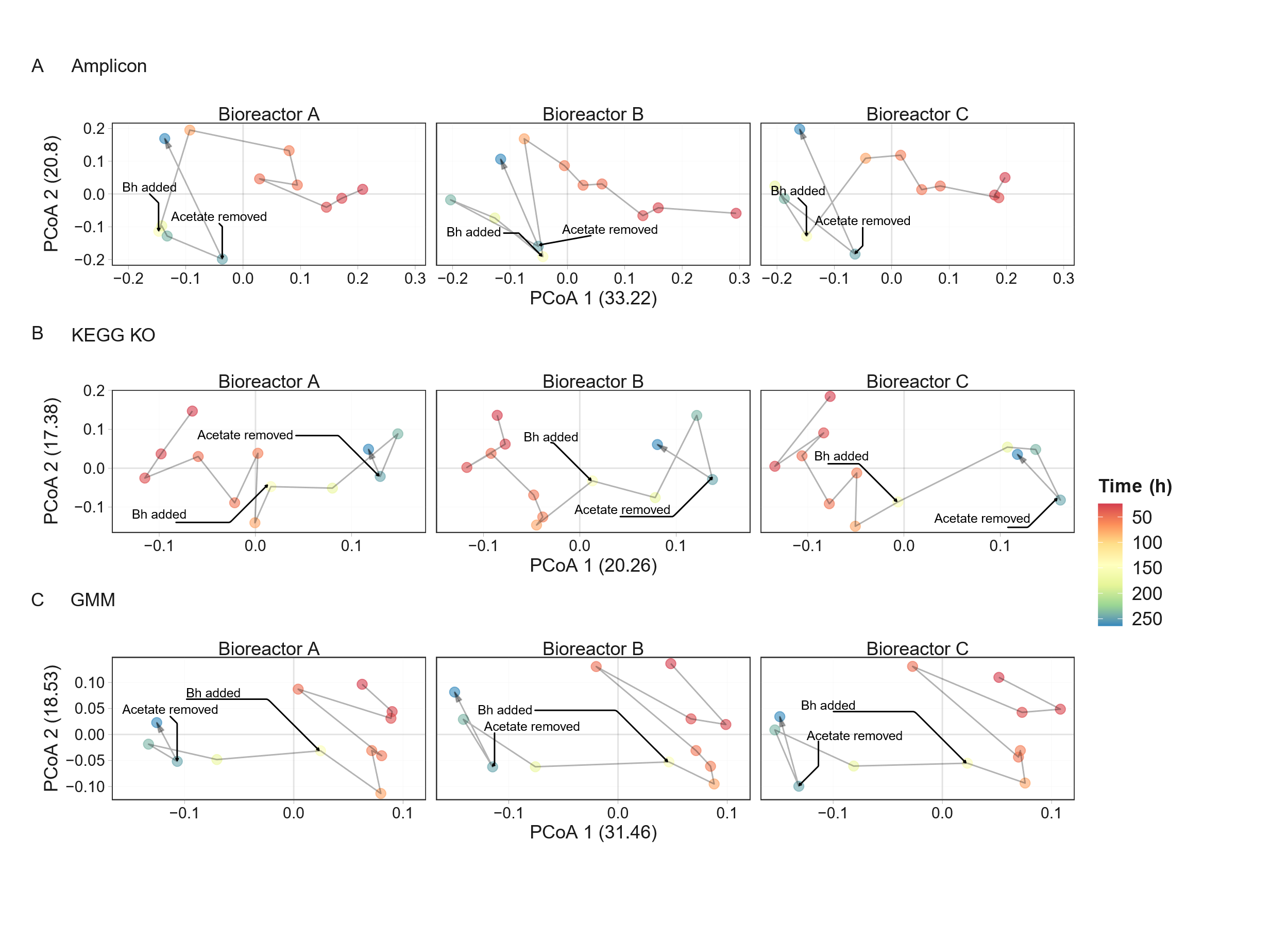


**Supplementary figure S9: Temporal compositional and transcriptional succession of MDb-MM.** Comparison of compositional and transcriptional community divergence. A) Community divergence based on Canberra distances using relative abundances of 16S rRNA gene. Only timepoints with metatranscriptomics data were analysed. B) Community convergence based on Canberra distances using relative expression of Kyoto Encyclopedia for Genes and Genomes (KEGG) ortholog. C) Community convergence based on Canberra distances using relative expression of Gut metabolic modules (GMMs). Each circle is labelled with corresponding timepoints for clarity.


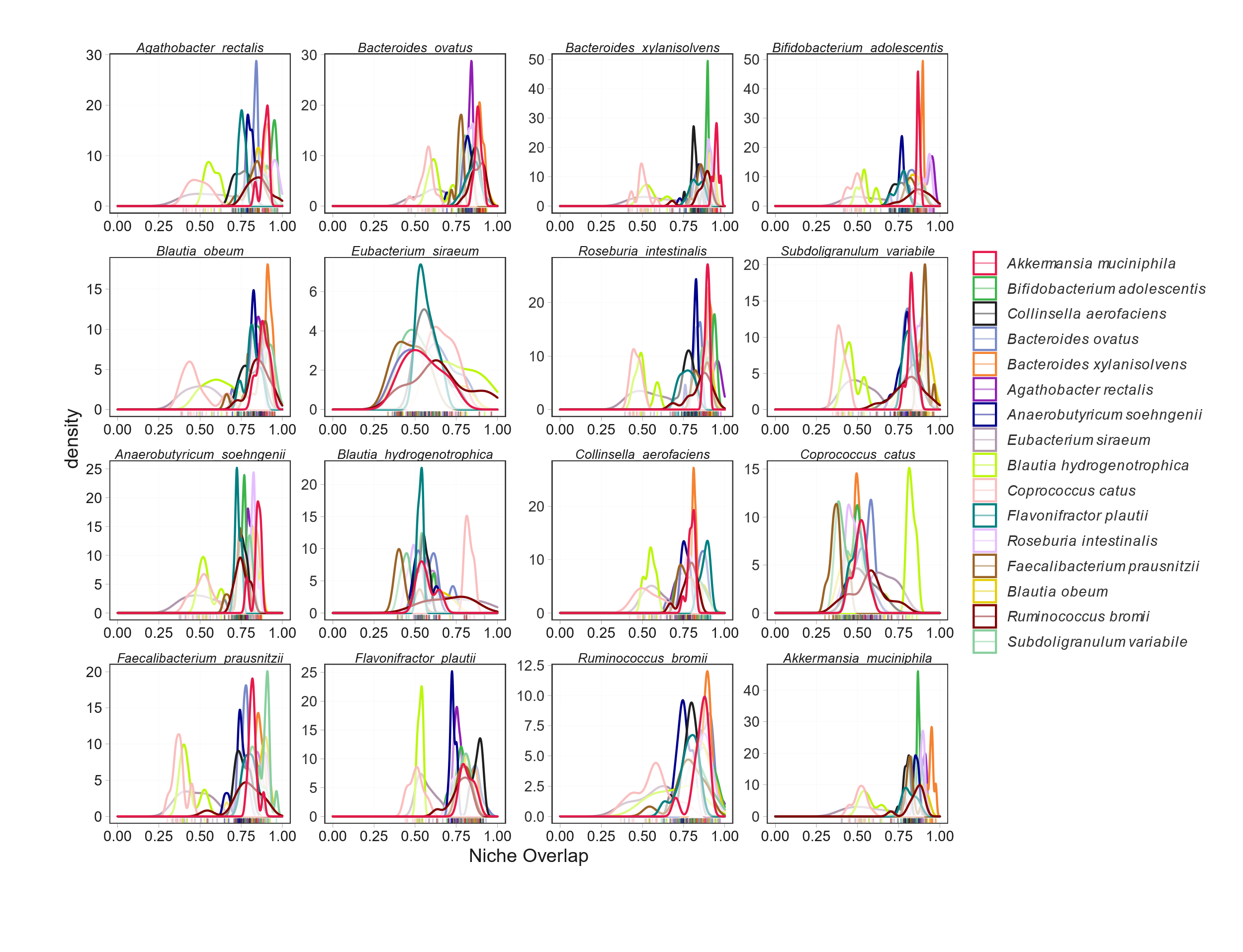


**Supplementary figure S10: Comparison of pair-wise niche overlap between species.** The niche overlap of each of the species was compared with other species in the community across timepoints. For details about niche overlap calculation are described in the methods section.


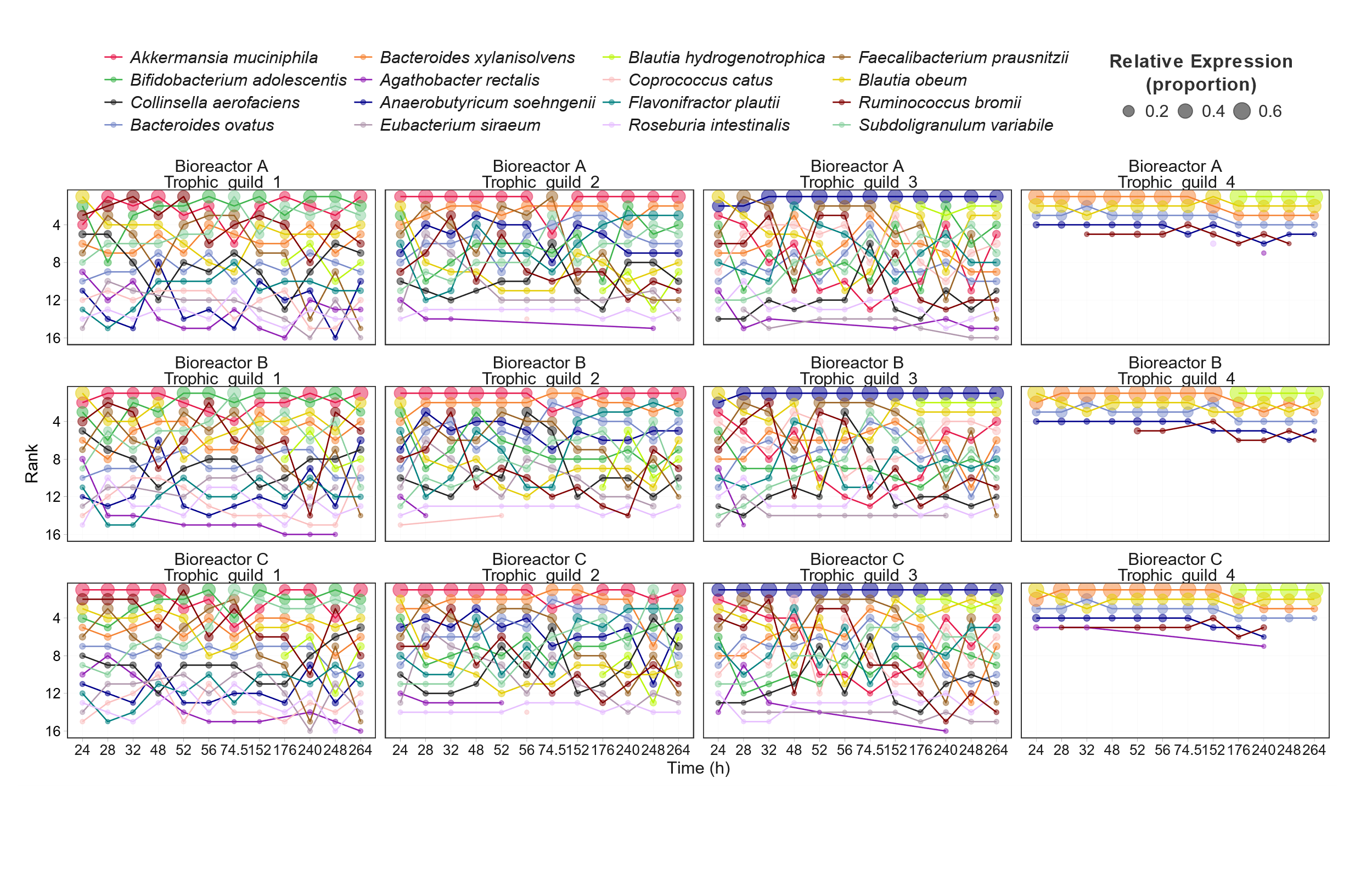


**Supplementary figure S11: Dynamic trophic roles of MDb-MM species.** For each trophic guild, each species was ranked based on their relative expression at each time point. The y-axis is ordered in reverse such that the high-ranking species is at the top and low-ranking species at the bottom. The size of the circle represents the relative expression of a species within a trophic guild.


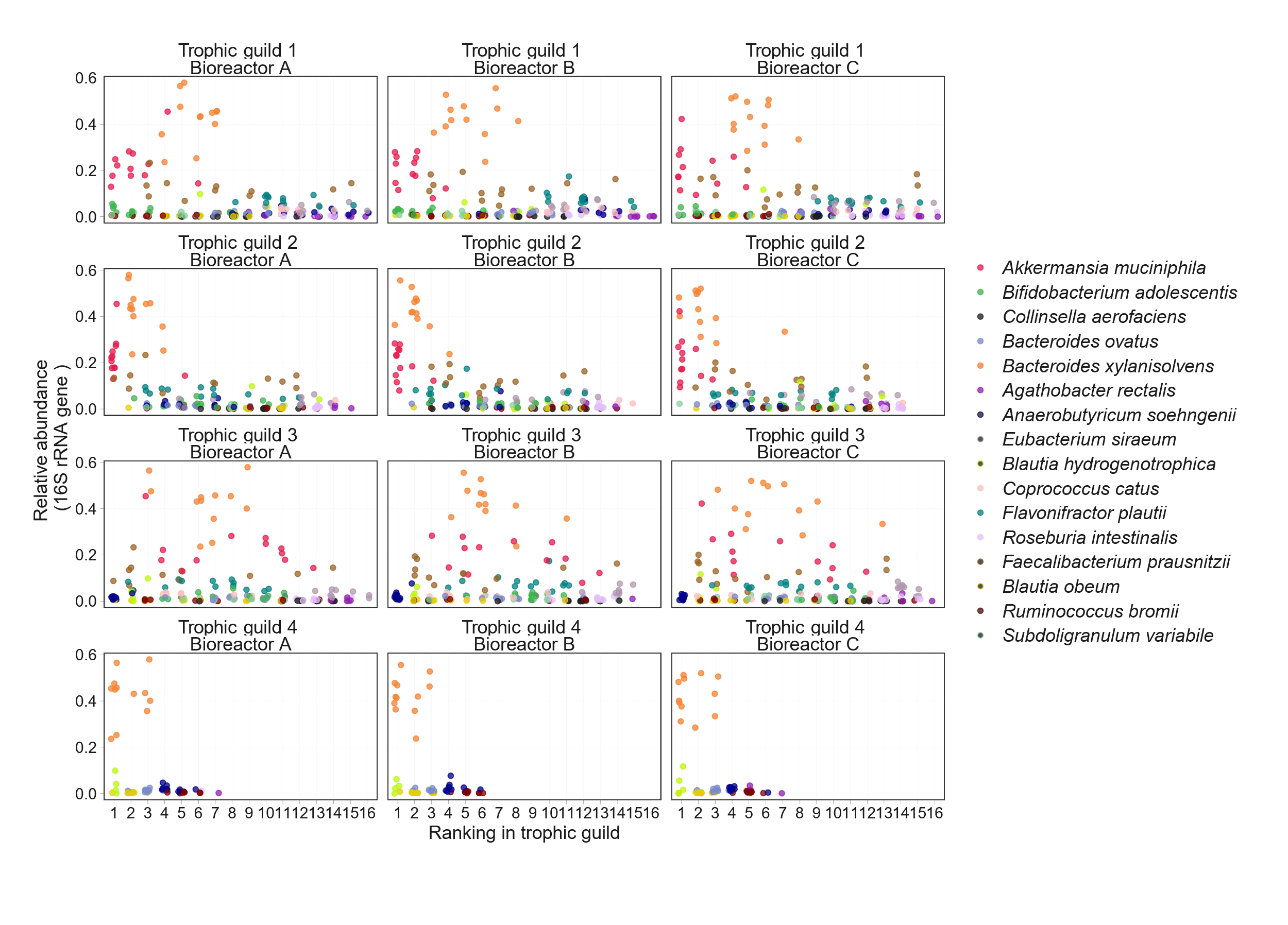


**Supplementary figure S12: Relationship between species relative abundance and its rank within a trophic guild.** Each circle represents 16S rRNA gene based relative abundance of the species at different time points and its rank within a trophic guild. For each trophic guild, each species was ranked based on their relative expression at each time point.


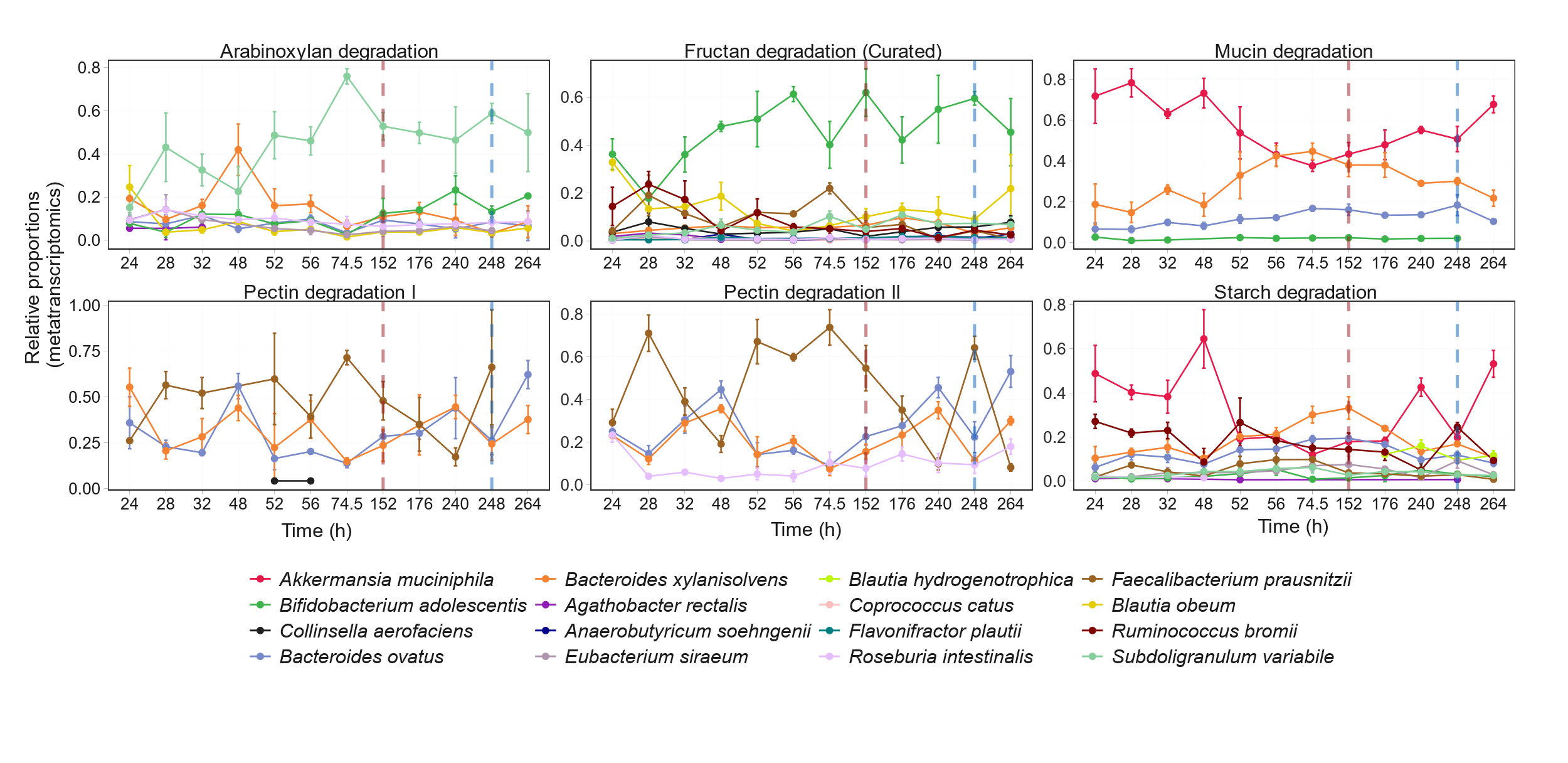


**Supplementary figure S13:** **Species specific contributions to complex substrate degradation (trophic guild 1)**. The relative proportions of species specific GMM expression are shown here. The vertical dashed red lines indicates introduction of *B. hydrogenotrophica*(152 h) and blue line indicates removal of acetate/feed change (248h).


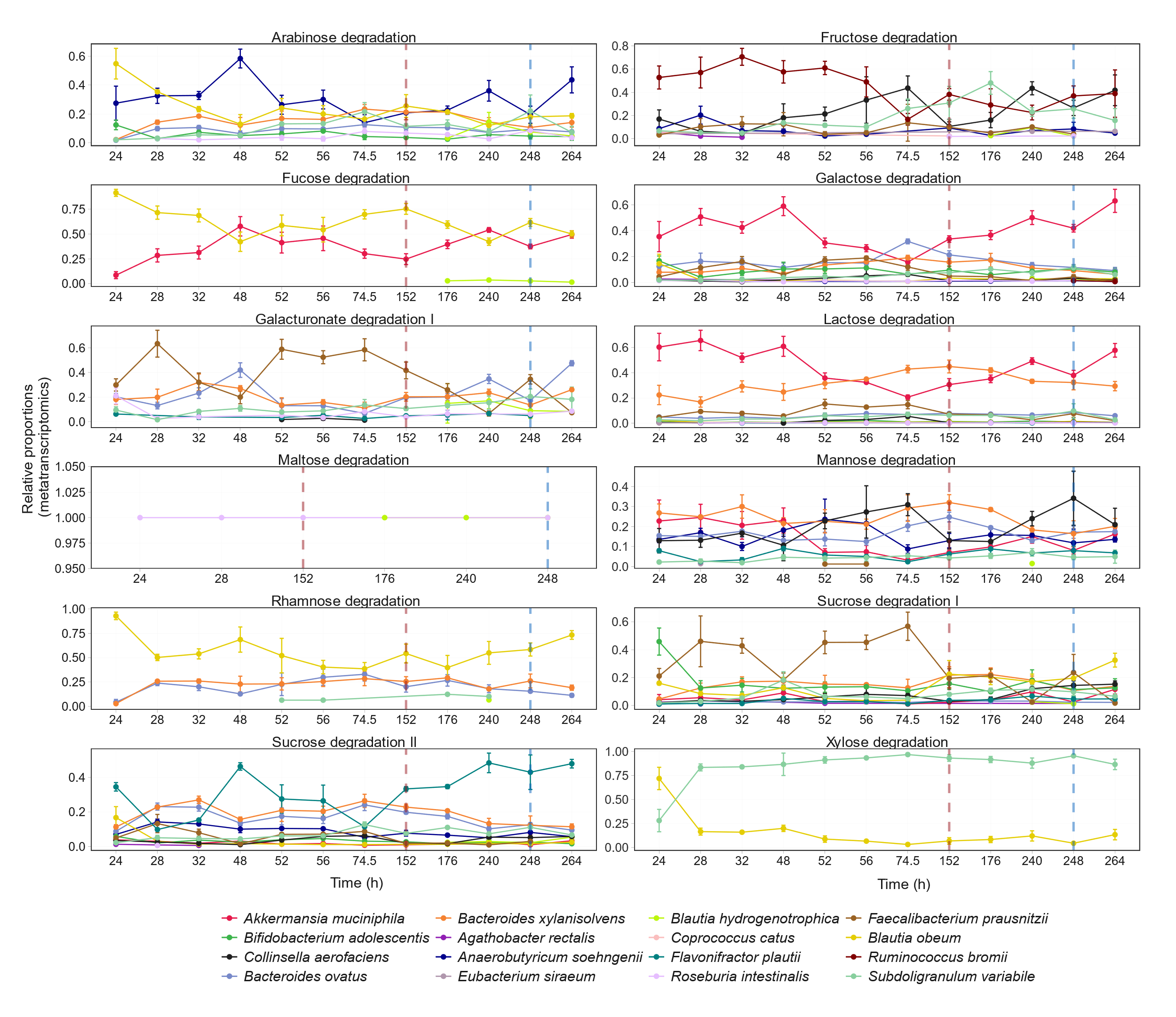


**Supplementary Figure S14:** **Species specific contributions to public goods (trophic guild 2)**. The relative proportions of species specific GMM expression are shown here. The vertical dashed red lines indicates introduction of *B. hydrogenotrophica*(152 h) and blue line indicates removal of acetate/feed change (248h).


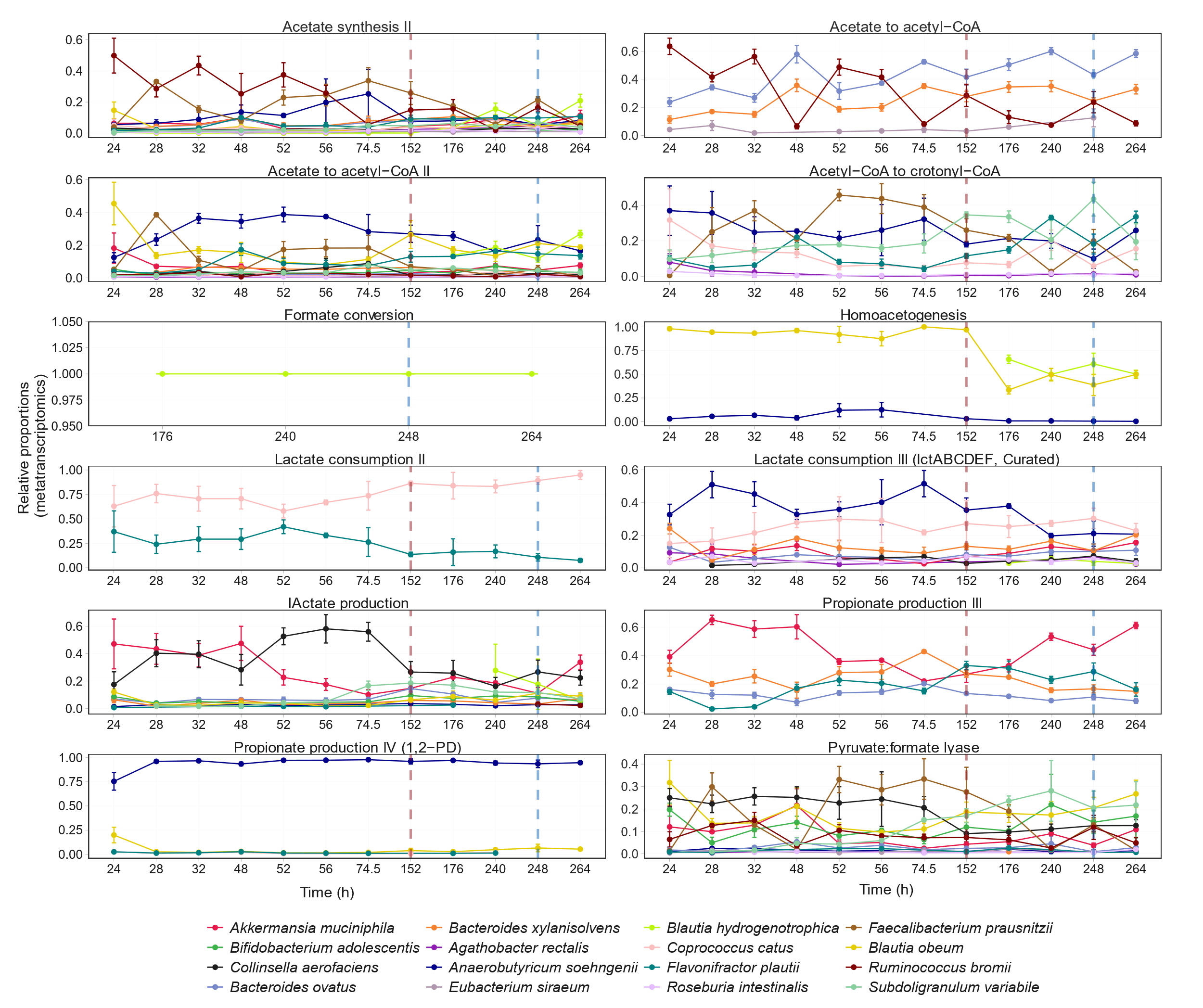


**Supplementary Figure S15:** **Species specific contributions to SCFA metabolism**. The relative proportions of species specific GMM expression are shown here. The vertical dashed red lines indicates introduction of *B. hydrogenotrophica*(152 h) and blue line indicates removal of acetate/feed change (248h).


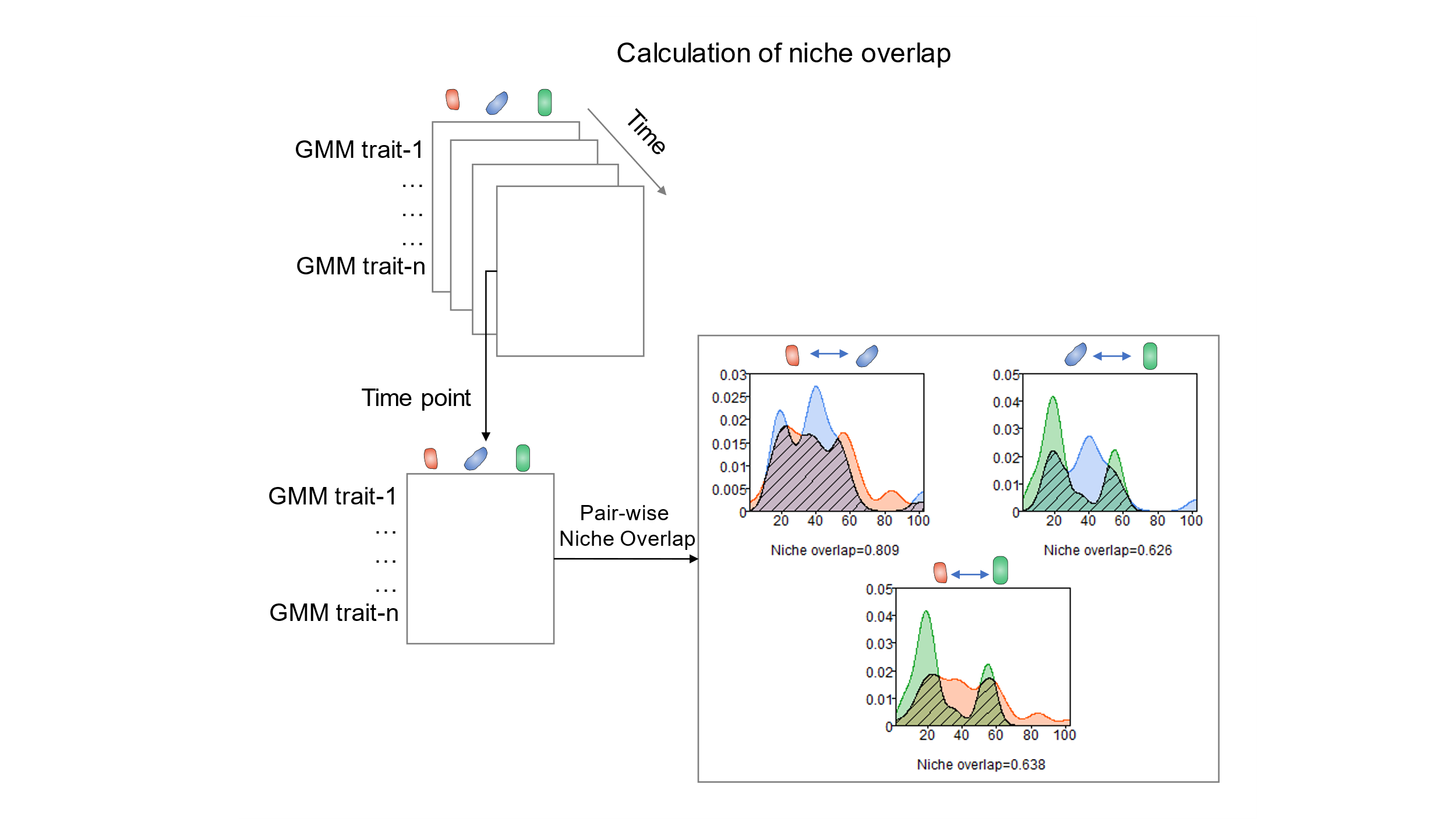


**Supplementary Figure 16: Schematic representation of pair-wise niche overlap calculation.** We used GMM trait expression values for calculating niche overlap. Only those GMM traits used for calculating trophic guild are used. A density distribution of traits is calculated for each species and overlap between the density distributions corresponding to the shaded are is estimated. This is done for all species pairs at each time point for every bioreactor separately.
